# Supplementary material for: Agromorphological Characterization of Quinoa (Chenopodium quinoa Willd.) Under Andean–Amazonian Region of Peru
Source: Plants (Basel). 2025 Dec 4;14(23):3689. doi: 10.3390/plants14233689 (PMC12693800; doi:10.3390/plants14233689)
Supplement: Supplementary file 1 [file plants-14-03689-s001.zip › ESM_1.html]

Agromorphological characterization of quinoa (Chenopodium quinoa Willd.) under Andean–Amazonian region of Peru


## Table of contents

- 1 Project Setup
- 2 Data import
- 3 Base de datos
- 4 Import data
- 5 Descriptors
- 6 Objective 1
  - 6.1 Study of the morphological diversity of qualitative variables in quinoa accessions
    - 6.1.1 Rasgos cualitativos (Na), Numero efectivo de clases(Ne), Diversidad genetica de Nei(He) y Shannon–Weaver diversity index (H′)
- 7 Objective 2
  - 7.1 Study of the morphological diversity of quantitative variables of quinoa accessions and variables with the greatest discriminant contribution
    - 7.1.1 Augmented design with entries as random effects and checks as fixed effects
- 8 Multivariate Analysis using BLUEs
- 9 Objective 3
- 10 Table blues

# Agromorphological characterization of quinoa (Chenopodium quinoa Willd.) under Andean–Amazonian region of Peru

Author

Victor-Hugo Baldera-Chapoñan; Germán De la Cruz; Segundo Oliva-Cruz1; Flavio Lozano-Isla

# 1 Project Setup

Code

```
library(factoextra)
library(vegan)
library(dplyr)
library(purrr)
library(writexl) 
source('https://inkaverse.com/setup.r')
```

```
Project directory:  C:/Users/HP/OneDrive/Documentos/git/Quinoa_diversity_vh 
CPU cores detected:  8 
CPU cores in use:  6
```

```
─ Session info ───────────────────────────────────────────────────────────────
 setting  value
 version  R version 4.5.1 (2025-06-13 ucrt)
 os       Windows 10 x64 (build 19045)
 system   x86_64, mingw32
 ui       RTerm
 language (EN)
 collate  Spanish_Peru.utf8
 ctype    Spanish_Peru.utf8
 tz       America/Lima
 date     2025-11-22
 pandoc   3.6.3 @ C:/Program Files/RStudio/resources/app/bin/quarto/bin/tools/ (via rmarkdown)
 quarto   NA @ C:\\Users\\HP\\AppData\\Local\\Programs\\Quarto\\bin\\quarto.exe

─ Packages ───────────────────────────────────────────────────────────────────
 package       * version  date (UTC) lib source
 abind           1.4-8    2024-09-12 [1] CRAN (R 4.5.0)
 agricolae       1.3-7    2023-10-22 [1] CRAN (R 4.5.0)
 AlgDesign       1.2.1.2  2025-04-06 [1] CRAN (R 4.5.0)
 askpass         1.2.1    2024-10-04 [1] CRAN (R 4.5.0)
 boot            1.3-32   2025-08-29 [1] CRAN (R 4.5.1)
 cachem          1.1.0    2024-05-16 [1] CRAN (R 4.5.0)
 car           * 3.1-3    2024-09-27 [1] CRAN (R 4.5.0)
 carData       * 3.0-5    2022-01-06 [1] CRAN (R 4.5.0)
 cellranger      1.1.0    2016-07-27 [1] CRAN (R 4.5.0)
 cli             3.6.5    2025-04-23 [1] CRAN (R 4.5.0)
 cluster         2.1.8.1  2025-03-12 [2] CRAN (R 4.5.1)
 codetools       0.2-20   2024-03-31 [2] CRAN (R 4.5.1)
 coro            1.1.0    2024-11-05 [1] CRAN (R 4.5.1)
 cowplot       * 1.2.0    2025-07-07 [1] CRAN (R 4.5.1)
 curl            7.0.0    2025-08-19 [1] CRAN (R 4.5.1)
 devtools      * 2.4.5    2022-10-11 [1] CRAN (R 4.5.0)
 digest          0.6.37   2024-08-19 [1] CRAN (R 4.5.0)
 dplyr         * 1.1.4    2023-11-17 [1] CRAN (R 4.5.0)
 DT              0.34.0   2025-09-02 [1] CRAN (R 4.5.1)
 ellipsis        0.3.2    2021-04-29 [1] CRAN (R 4.5.0)
 ellmer          0.3.2    2025-09-03 [1] CRAN (R 4.5.1)
 emmeans       * 1.11.2-8 2025-08-27 [1] CRAN (R 4.5.1)
 estimability    1.5.1    2024-05-12 [1] CRAN (R 4.5.0)
 evaluate        1.0.5    2025-08-27 [1] CRAN (R 4.5.1)
 factoextra    * 1.0.7    2020-04-01 [1] CRAN (R 4.5.0)
 FactoMineR    * 2.12     2025-07-23 [1] CRAN (R 4.5.1)
 farver          2.1.2    2024-05-13 [1] CRAN (R 4.5.0)
 fastmap         1.2.0    2024-05-15 [1] CRAN (R 4.5.0)
 flashClust      1.01-2   2012-08-21 [1] CRAN (R 4.5.0)
 forcats       * 1.0.0    2023-01-29 [1] CRAN (R 4.5.0)
 Formula         1.2-5    2023-02-24 [1] CRAN (R 4.5.0)
 fs              1.6.6    2025-04-12 [1] CRAN (R 4.5.0)
 gargle          1.6.0    2025-09-03 [1] CRAN (R 4.5.1)
 generics        0.1.4    2025-05-09 [1] CRAN (R 4.5.1)
 ggplot2       * 4.0.0    2025-09-11 [1] CRAN (R 4.5.1)
 ggrepel         0.9.6    2024-09-07 [1] CRAN (R 4.5.0)
 glue            1.8.0    2024-09-30 [1] CRAN (R 4.5.0)
 googledrive   * 2.1.2    2025-09-10 [1] CRAN (R 4.5.1)
 googlesheets4 * 1.1.2    2025-09-03 [1] CRAN (R 4.5.1)
 gtable          0.3.6    2024-10-25 [1] CRAN (R 4.5.0)
 hms             1.1.3    2023-03-21 [1] CRAN (R 4.5.0)
 htmltools       0.5.8.1  2024-04-04 [1] CRAN (R 4.5.0)
 htmlwidgets     1.6.4    2023-12-06 [1] CRAN (R 4.5.0)
 httpuv          1.6.16   2025-04-16 [1] CRAN (R 4.5.0)
 httr            1.4.7    2023-08-15 [1] CRAN (R 4.5.0)
 httr2           1.2.1    2025-07-22 [1] CRAN (R 4.5.1)
 huito         * 0.2.6    2025-10-18 [1] CRAN (R 4.5.1)
 inti          * 0.6.8    2025-09-13 [1] Github (flavjack/inti@a0187d8)
 jsonlite        2.0.0    2025-03-27 [1] CRAN (R 4.5.0)
 knitr         * 1.50     2025-03-16 [1] CRAN (R 4.5.0)
 later           1.4.4    2025-08-27 [1] CRAN (R 4.5.1)
 lattice         0.22-7   2025-04-02 [2] CRAN (R 4.5.1)
 leaps           3.2      2024-06-10 [1] CRAN (R 4.5.0)
 lifecycle       1.0.4    2023-11-07 [1] CRAN (R 4.5.0)
 lme4          * 1.1-37   2025-03-26 [1] CRAN (R 4.5.0)
 lubridate     * 1.9.4    2024-12-08 [1] CRAN (R 4.5.0)
 magick        * 2.9.0    2025-09-08 [1] CRAN (R 4.5.1)
 magrittr        2.0.3    2022-03-30 [1] CRAN (R 4.5.1)
 MASS          * 7.3-65   2025-02-28 [2] CRAN (R 4.5.1)
 Matrix        * 1.7-4    2025-08-28 [1] CRAN (R 4.5.1)
 memoise         2.0.1    2021-11-26 [1] CRAN (R 4.5.0)
 mgcv            1.9-3    2025-04-04 [2] CRAN (R 4.5.1)
 mime            0.13     2025-03-17 [1] CRAN (R 4.5.0)
 miniUI          0.1.2    2025-04-17 [1] CRAN (R 4.5.0)
 minqa           1.2.8    2024-08-17 [1] CRAN (R 4.5.0)
 mnormt          2.1.1    2022-09-26 [1] CRAN (R 4.5.0)
 multcomp      * 1.4-28   2025-01-29 [1] CRAN (R 4.5.0)
 multcompView    0.1-10   2024-03-08 [1] CRAN (R 4.5.0)
 mvtnorm       * 1.3-3    2025-01-10 [1] CRAN (R 4.5.0)
 nlme            3.1-168  2025-03-31 [2] CRAN (R 4.5.1)
 nloptr          2.2.1    2025-03-17 [1] CRAN (R 4.5.0)
 openssl         2.3.3    2025-05-26 [1] CRAN (R 4.5.1)
 permute       * 0.9-8    2025-06-25 [1] CRAN (R 4.5.1)
 pillar          1.11.1   2025-09-17 [1] CRAN (R 4.5.1)
 pkgbuild        1.4.8    2025-05-26 [1] CRAN (R 4.5.1)
 pkgconfig       2.0.3    2019-09-22 [1] CRAN (R 4.5.0)
 pkgload         1.4.0    2024-06-28 [1] CRAN (R 4.5.0)
 profvis         0.4.0    2024-09-20 [1] CRAN (R 4.5.0)
 promises        1.3.3    2025-05-29 [1] CRAN (R 4.5.1)
 psych         * 2.5.6    2025-06-23 [1] CRAN (R 4.5.1)
 purrr         * 1.1.0    2025-07-10 [1] CRAN (R 4.5.1)
 R6              2.6.1    2025-02-15 [1] CRAN (R 4.5.0)
 rappdirs        0.3.3    2021-01-31 [1] CRAN (R 4.5.0)
 rbibutils       2.3      2024-10-04 [1] CRAN (R 4.5.0)
 RColorBrewer    1.1-3    2022-04-03 [1] CRAN (R 4.5.0)
 Rcpp            1.1.0    2025-07-02 [1] CRAN (R 4.5.1)
 Rdpack          2.6.4    2025-04-09 [1] CRAN (R 4.5.0)
 readr         * 2.1.5    2024-01-10 [1] CRAN (R 4.5.0)
 reformulas      0.4.1    2025-04-30 [1] CRAN (R 4.5.0)
 remotes         2.5.0    2024-03-17 [1] CRAN (R 4.5.0)
 RhpcBLASctl   * 0.23-42  2023-02-11 [1] CRAN (R 4.5.0)
 rlang           1.1.6    2025-04-11 [1] CRAN (R 4.5.0)
 rmarkdown       2.30     2025-09-28 [1] CRAN (R 4.5.1)
 rstudioapi      0.17.1   2024-10-22 [1] CRAN (R 4.5.0)
 S7              0.2.0    2024-11-07 [1] CRAN (R 4.5.1)
 sandwich        3.1-1    2024-09-15 [1] CRAN (R 4.5.0)
 scales          1.4.0    2025-04-24 [1] CRAN (R 4.5.0)
 scatterplot3d   0.3-44   2023-05-05 [1] CRAN (R 4.5.0)
 sessioninfo   * 1.2.3    2025-02-05 [1] CRAN (R 4.5.0)
 shiny         * 1.11.1   2025-07-03 [1] CRAN (R 4.5.1)
 showtext        0.9-7    2024-03-02 [1] CRAN (R 4.5.0)
 showtextdb      3.0      2020-06-04 [1] CRAN (R 4.5.0)
 stringi         1.8.7    2025-03-27 [1] CRAN (R 4.5.0)
 stringr       * 1.5.2    2025-09-08 [1] CRAN (R 4.5.1)
 survival      * 3.8-3    2024-12-17 [2] CRAN (R 4.5.1)
 sysfonts        0.8.9    2024-03-02 [1] CRAN (R 4.5.0)
 TH.data       * 1.1-4    2025-09-02 [1] CRAN (R 4.5.1)
 tibble        * 3.3.0    2025-06-08 [1] CRAN (R 4.5.1)
 tidyr         * 1.3.1    2024-01-24 [1] CRAN (R 4.5.0)
 tidyselect      1.2.1    2024-03-11 [1] CRAN (R 4.5.0)
 tidyverse     * 2.0.0    2023-02-22 [1] CRAN (R 4.5.0)
 timechange      0.3.0    2024-01-18 [1] CRAN (R 4.5.0)
 tzdb            0.5.0    2025-03-15 [1] CRAN (R 4.5.0)
 urlchecker      1.0.1    2021-11-30 [1] CRAN (R 4.5.0)
 usethis       * 3.2.1    2025-09-06 [1] CRAN (R 4.5.1)
 vctrs           0.6.5    2023-12-01 [1] CRAN (R 4.5.0)
 vegan         * 2.7-1    2025-06-05 [1] CRAN (R 4.5.1)
 withr           3.0.2    2024-10-28 [1] CRAN (R 4.5.0)
 writexl       * 1.5.4    2025-04-15 [1] CRAN (R 4.5.0)
 xfun            0.53     2025-08-19 [1] CRAN (R 4.5.1)
 xtable          1.8-4    2019-04-21 [1] CRAN (R 4.5.0)
 yaml            2.3.10   2024-07-26 [1] CRAN (R 4.5.0)
 zoo             1.8-14   2025-04-10 [1] CRAN (R 4.5.0)

 [1] C:/Users/HP/AppData/Local/R/win-library/4.5
 [2] C:/Program Files/R/R-4.5.1/library
 * ── Packages attached to the search path.

──────────────────────────────────────────────────────────────────────────────
```

# 2 Data import

# 3 Base de datos

> https://docs.google.com/spreadsheets/d/1Rq304iX6UYhdvwDt7oaQHAIpIgtWKvEJb3WreFOw0Fw/edit?gid=1506576414#gid=1506576414

# 4 Import data

Code

```
url <- "https://docs.google.com/spreadsheets/d/1Rq304iX6UYhdvwDt7oaQHAIpIgtWKvEJb3WreFOw0Fw/edit?gid=1506576414#gid=1506576414"

gs <- as_sheets_id(url)

fb <- range_read(gs, sheet = "fb") %>% 
  mutate(across(cdpf_67_1:ncol(.), ~ as.character(.))) %>% 
  pivot_longer(!c(1:TREATMENT)) %>% 
  separate(name, c("name", "pheno", "sample")) %>% 
  dplyr::select(!c(pheno)) %>% 
  pivot_wider() %>% 
  mutate(across(c(1:sample), as.factor)) %>% 
  as.data.frame()

str(fb)
## 'data.frame':    1030 obs. of  36 variables:
##  $ PLOT     : Factor w/ 162 levels "0011","1001",..: 5 5 5 5 5 6 6 6 6 6 ...
##  $ CODIGO   : Factor w/ 162 levels "CH1","CH2","CH3",..: 8 8 8 8 8 9 9 9 9 9 ...
##  $ NUM      : Factor w/ 159 levels "0011","10","100",..: 91 91 91 91 91 102 102 102 102 102 ...
##  $ EXPT     : Factor w/ 1 level "quinoaGB": 1 1 1 1 1 1 1 1 1 1 ...
##  $ LOCATION : Factor w/ 1 level "1": 1 1 1 1 1 1 1 1 1 1 ...
##  $ YEAR     : Factor w/ 1 level "2025": 1 1 1 1 1 1 1 1 1 1 ...
##  $ ROW      : Factor w/ 16 levels "1","10","11",..: 1 1 1 1 1 1 1 1 1 1 ...
##  $ COLUMN   : Factor w/ 13 levels "1","10","11",..: 8 8 8 8 8 9 9 9 9 9 ...
##  $ CHECKS   : Factor w/ 2 levels "0","1": 1 1 1 1 1 1 1 1 1 1 ...
##  $ BLOCK    : Factor w/ 16 levels "1","10","11",..: 1 1 1 1 1 1 1 1 1 1 ...
##  $ ENTRY    : Factor w/ 158 levels "1","10","100",..: 93 93 93 93 93 104 104 104 104 104 ...
##  $ TREATMENT: Factor w/ 158 levels "CH1","CH2","CH3",..: 93 93 93 93 93 104 104 104 104 104 ...
##  $ sample   : Factor w/ 5 levels "1","2","3","4",..: 1 2 3 4 5 1 2 3 4 5 ...
##  $ cdpf     : chr  "1 Green" NA NA NA ...
##  $ frp      : chr  "1 Glomerular" NA NA NA ...
##  $ ndh50f   : chr  "76" NA NA NA ...
##  $ spad     : chr  "44.8" "43.3" "44.3" NA ...
##  $ pse      : chr  "Yes" NA NA NA ...
##  $ ppfi     : chr  "100" NA NA NA ...
##  $ cpmf     : chr  "5 Yellow" NA NA NA ...
##  $ cpmp     : chr  "content://com.android.externalstorage.documents/tree/primary%3Aceigras/document/primary%3Aceigras%2Fplot_data%2"| __truncated__ NA NA NA ...
##  $ lgtp     : chr  "30.5" "40" "30" "43" ...
##  $ dtp      : chr  "8.66" "8.14" "8.05" "6.3" ...
##  $ dsp      : chr  "1 Laxa" NA NA NA ...
##  $ ndh50mf  : chr  "90" NA NA NA ...
##  $ acm      : chr  "No" NA NA NA ...
##  $ alp      : chr  "97.5" NA NA NA ...
##  $ dmt      : chr  "1.6" "1.33" "1" NA ...
##  $ hbc      : chr  "3 Branched up to the middle third" NA NA NA ...
##  $ ctp      : chr  "5 Yellow" NA NA NA ...
##  $ gdh      : chr  "1 Light" NA NA NA ...
##  $ palp10   : chr  "1.95" NA NA NA ...
##  $ ps10     : chr  "638.26" NA NA NA ...
##  $ p100g    : chr  "0.34" NA NA NA ...
##  $ cepp     : chr  "2 White" NA NA NA ...
##  $ fmg      : chr  "2 Cilíndrico" NA NA NA ...

quali <- fb %>% 
  dplyr::select(c(1:sample, cdpf, frp, cpmf, dsp, hbc, ctp, pse,  gdh, acm, cepp, fmg)) %>% 
  drop_na(frp) %>% 
  filter(!str_detect(CODIGO, "CH"))

str(quali)
## 'data.frame':    136 obs. of  24 variables:
##  $ PLOT     : Factor w/ 162 levels "0011","1001",..: 5 6 7 8 9 10 11 12 13 14 ...
##  $ CODIGO   : Factor w/ 162 levels "CH1","CH2","CH3",..: 8 9 10 11 12 13 14 15 16 17 ...
##  $ NUM      : Factor w/ 159 levels "0011","10","100",..: 91 102 113 124 135 146 2 13 24 35 ...
##  $ EXPT     : Factor w/ 1 level "quinoaGB": 1 1 1 1 1 1 1 1 1 1 ...
##  $ LOCATION : Factor w/ 1 level "1": 1 1 1 1 1 1 1 1 1 1 ...
##  $ YEAR     : Factor w/ 1 level "2025": 1 1 1 1 1 1 1 1 1 1 ...
##  $ ROW      : Factor w/ 16 levels "1","10","11",..: 1 1 1 1 1 1 1 1 1 1 ...
##  $ COLUMN   : Factor w/ 13 levels "1","10","11",..: 8 9 10 11 12 3 4 5 4 3 ...
##  $ CHECKS   : Factor w/ 2 levels "0","1": 1 1 1 1 1 1 1 1 1 1 ...
##  $ BLOCK    : Factor w/ 16 levels "1","10","11",..: 1 1 1 1 1 1 1 1 1 1 ...
##  $ ENTRY    : Factor w/ 158 levels "1","10","100",..: 93 104 115 126 137 148 2 13 24 35 ...
##  $ TREATMENT: Factor w/ 158 levels "CH1","CH2","CH3",..: 93 104 115 126 137 148 4 15 26 37 ...
##  $ sample   : Factor w/ 5 levels "1","2","3","4",..: 1 1 1 1 1 1 1 1 1 1 ...
##  $ cdpf     : chr  "1 Green" "2 PÃºrpura" "1 Green" "2 PÃºrpura" ...
##  $ frp      : chr  "1 Glomerular" "1 Glomerular" "1 Glomerular" "1 Glomerular" ...
##  $ cpmf     : chr  "5 Yellow" "11 Red y Pink" "5 Yellow" "6 Orange" ...
##  $ dsp      : chr  "1 Laxa" "2 Intermedia" "1 Laxa" "2 Intermedia" ...
##  $ hbc      : chr  "3 Branched up to the middle third" "3 Branched up to the middle third" "3 Branched up to the middle third" "2 Branched up to the lower third" ...
##  $ ctp      : chr  "5 Yellow" "2 PÃºrpura" "10 Green" "4 Pink" ...
##  $ pse      : chr  "Yes" "Yes" "Yes" "Yes" ...
##  $ gdh      : chr  "1 Light" "1 Light" "2 Regular" "1 Light" ...
##  $ acm      : chr  "No" "No" "No" "Yes" ...
##  $ cepp     : chr  "2 White" "4 Café claro" "3 Cream" "3 Cream" ...
##  $ fmg      : chr  "2 Cilíndrico" "1 Lenticular" "2 Cilíndrico" "2 Cilíndrico" ...

quali <- quali %>%
  mutate(across(
    c( cdpf, frp, cpmf, dsp, hbc, ctp, pse,  gdh, acm, cepp, fmg),  
    ~ gsub("^\\d+\\s*", "", .)  
  )) %>%
  mutate(across(
    c( cdpf, frp, cpmf, dsp, hbc, ctp, pse,  gdh, acm, cepp, fmg),  
    ~ iconv(., from = "UTF-8", to = "ASCII//TRANSLIT")  
  )) %>%
  mutate(across(
    c( cdpf, frp, cpmf, dsp, hbc, ctp, pse,  gdh, acm, cepp, fmg),
    ~ str_replace_all(., 
                      c("Ãº" = "ú", 
                        "Ã³" = "ó", 
                        "Ã" = "Á", 
                        "A3" = "o", 
                        "PAorpura" = "Púrpura", 
                        "Mixtura (pAorpura y rojo)" = "Mixtura (Púrpura y rojo)", 
                        "FALSO" = "Falso",
                        "VERDADERO" = "Verdadero",
                        "MarrA3n" = "Marrón"))
  ))


head(quali)
##   PLOT      CODIGO NUM     EXPT LOCATION YEAR ROW COLUMN CHECKS BLOCK ENTRY
## 1 1004 UNTRM121004   4 quinoaGB        1 2025   1      4      0     1     4
## 2 1005 UNTRM121005   5 quinoaGB        1 2025   1      5      0     1     5
## 3 1006 UNTRM121006   6 quinoaGB        1 2025   1      6      0     1     6
## 4 1007 UNTRM121007   7 quinoaGB        1 2025   1      7      0     1     7
## 5 1008 UNTRM121008   8 quinoaGB        1 2025   1      8      0     1     8
## 6 1009 UNTRM121009   9 quinoaGB        1 2025   1     11      0     1     9
##   TREATMENT sample    cdpf        frp       cpmf        dsp
## 1        G4      1   Green Glomerular     Yellow       Laxa
## 2        G5      1 Púrpura Glomerular Red y Pink Intermedia
## 3        G6      1   Green Glomerular     Yellow       Laxa
## 4        G7      1 Púrpura Glomerular     Orange Intermedia
## 5        G8      1 Púrpura Intermedia    Púrpura Intermedia
## 6        G9      1   Green Glomerular     Yellow       Laxa
##                               hbc     ctp pse     gdh acm       cepp        fmg
## 1 Branched up to the middle third  Yellow Yes   Light  No      White Cilindrico
## 2 Branched up to the middle third Púrpura Yes   Light  No Cafe claro Lenticular
## 3 Branched up to the middle third   Green Yes Regular  No      Cream Cilindrico
## 4  Branched up to the lower third    Pink Yes   Light Yes      Cream Cilindrico
## 5 Branched up to the middle third   Green Yes   Light Yes      Cream Cilindrico
## 6 Branched up to the middle third   Green Yes   Light  No      Cream Cilindrico

quali %>% web_table()
```

# 5 Descriptors

Code

```
info <- range_read(gs, sheet = "descriptor") %>%
  rename_with(~ iconv(.x, from = "UTF-8", to = "ASCII//TRANSLIT")) %>%
  rename_with(~ str_replace_all(.x, "^[^a-zA-Z0-9]+|[^a-zA-Z0-9]+$", "")) %>%
  tidyr::fill(c(abbreviatio_sp , abbreviatio_en, descriptors), .direction = "down")

info %>% str()
## tibble [23 × 3] (S3: tbl_df/tbl/data.frame)
##  $ descriptors   : chr [1:23] "Panicle color at 50% flowering" "Panicle color at 50% physiological maturity" "Panicle shape" "Panicle length (cm)" ...
##  $ abbreviatio_sp: chr [1:23] "cdpf" "cpmf" "frp" "lgtp" ...
##  $ abbreviatio_en: chr [1:23] "PCF" "PCM" "PSH" "PL" ...
```

# 6 Objective 1

## 6.1 Study of the morphological diversity of qualitative variables in quinoa accessions

### 6.1.1 Rasgos cualitativos (Na), Numero efectivo de clases(Ne), Diversidad genetica de Nei(He) y Shannon–Weaver diversity index (H′)

Code

```
quali_traits <- quali %>%
  dplyr::select(cdpf, frp, cpmf, dsp, hbc, ctp, pse, gdh, acm, cepp, fmg)

trait_vars <- names(quali_traits)

trait_labels <- info %>%
  filter(abbreviatio_sp %in% trait_vars) %>%
  dplyr::select(abbreviatio_sp, descriptors) %>%
  deframe()

summarise_trait <- function(df, var, label) {
  tab <- df %>%
    count(.data[[var]], name = "Na") %>%
    mutate(fi = Na / sum(Na))
  
  Nc     <- nrow(tab)
  sum_p2 <- sum(tab$fi^2)
  Ne     <- 1 / sum_p2
  He     <- sum_p2
  Hprime <- diversity(tab$Na, index = "shannon")
  
  tab %>%
    mutate(
      `Qualitative trait` = label,
      Nc     = Nc,
      Ne     = Ne,
      He     = He,
      Hprime = Hprime
    ) %>%
    rename(`Phenotypic classes observed` = !!sym(var)) %>%
    dplyr::select(
      `Qualitative trait`,
      `Phenotypic classes observed`,
      Na, fi, Nc, Ne, He, Hprime
    )
}

qual_table <- map_dfr(
  trait_vars,
  ~ summarise_trait(quali, var = .x, label = trait_labels[.x])
)

trait_summary <- qual_table %>%
  distinct(`Qualitative trait`, Nc, Ne, He, Hprime)

summary_mean_se <- trait_summary %>%
  summarise(
    Nc_mean     = mean(Nc),
    Nc_se       = sd(Nc) / sqrt(n()),
    Ne_mean     = mean(Ne),
    Ne_se       = sd(Ne) / sqrt(n()),
    He_mean     = mean(He),
    He_se       = sd(He) / sqrt(n()),
    Hprime_mean = mean(Hprime),
    Hprime_se   = sd(Hprime) / sqrt(n())
  )

qual_table_out <- qual_table %>%
  mutate(across(c(fi, Nc, Ne, He, Hprime), ~ round(., 2))) %>%
  rename(`H′` = Hprime)

qual_table_fmt <- qual_table_out %>%
  group_by(`Qualitative trait`) %>%
  mutate(
    Na  = as.character(Na),
    fi  = sprintf("%.2f", fi),
    Nc  = if_else(row_number() == 1, sprintf("%.2f", Nc), ""),
    Ne  = if_else(row_number() == 1, sprintf("%.2f", Ne), ""),
    He  = if_else(row_number() == 1, sprintf("%.2f", He), ""),
    `H′` = if_else(row_number() == 1, sprintf("%.2f", `H′`), ""),
    `Qualitative trait` = if_else(
      row_number() == 1,
      `Qualitative trait`,
      ""                        
    )
  ) %>%
  ungroup()

mean_row_formatted <- summary_mean_se %>%
  transmute(
    `Qualitative trait`            = "Mean ± SE",
    `Phenotypic classes observed` = "",
    Na  = "",
    fi  = "",
    Nc  = sprintf("%.2f ± %.2f", Nc_mean, Nc_se),
    Ne  = sprintf("%.2f ± %.2f", Ne_mean, Ne_se),
    He  = sprintf("%.2f ± %.2f", He_mean, He_se),
    `H′`= sprintf("%.2f ± %.2f", Hprime_mean, Hprime_se)
  )

Diversity <- qual_table_fmt %>%
  mutate(across(c(Nc, Ne, He, `H′`), as.character)) %>%
  bind_rows(mean_row_formatted)

Diversity %>%
  knitr::kable(caption = "Shannon–Weaver diversity index (H′)")
```

Shannon–Weaver diversity index (H′)


| Qualitative trait | Phenotypic classes observed | Na | fi | Nc | Ne | He | H′ |
| --- | --- | --- | --- | --- | --- | --- | --- |
| Panicle color at 50% flowering | Green | 52 | 0.38 | 4.00 | 2.64 | 0.38 | 1.11 |
|  | Mixtura (pAorpura y Red) | 9 | 0.07 |  |  |  |  |
|  | Púrpura | 64 | 0.47 |  |  |  |  |
|  | Red | 11 | 0.08 |  |  |  |  |
| Panicle shape | Amarantiform | 7 | 0.05 | 3.00 | 1.65 | 0.60 | 0.69 |
|  | Glomerular | 102 | 0.75 |  |  |  |  |
|  | Intermedia | 27 | 0.20 |  |  |  |  |
| Panicle color at 50% physiological maturity | Green | 10 | 0.07 | 10.00 | 4.71 | 0.21 | 1.79 |
|  | Grey | 1 | 0.01 |  |  |  |  |
|  | Marron | 2 | 0.01 |  |  |  |  |
|  | Orange | 30 | 0.22 |  |  |  |  |
|  | Pink | 19 | 0.14 |  |  |  |  |
|  | Púrpura | 10 | 0.07 |  |  |  |  |
|  | Red y Pink | 12 | 0.09 |  |  |  |  |
|  | Red y Yellow | 1 | 0.01 |  |  |  |  |
|  | White | 3 | 0.02 |  |  |  |  |
|  | Yellow | 48 | 0.35 |  |  |  |  |
| Panicle density | Compact | 6 | 0.04 | 3.00 | 1.43 | 0.70 | 0.57 |
|  | Intermedia | 18 | 0.13 |  |  |  |  |
|  | Laxa | 112 | 0.82 |  |  |  |  |
| Growth habit | Branched up to the lower third | 24 | 0.18 | 4.00 | 2.25 | 0.44 | 1.01 |
|  | Branched up to the middle third | 84 | 0.62 |  |  |  |  |
|  | Branched with an undefined main panicle | 4 | 0.03 |  |  |  |  |
|  | Simple | 24 | 0.18 |  |  |  |  |
| Main stem color | Green | 57 | 0.42 | 9.00 | 4.20 | 0.24 | 1.76 |
|  | Grey | 3 | 0.02 |  |  |  |  |
|  | Marron | 5 | 0.04 |  |  |  |  |
|  | Orange | 7 | 0.05 |  |  |  |  |
|  | Pink | 7 | 0.05 |  |  |  |  |
|  | Púrpura | 21 | 0.15 |  |  |  |  |
|  | Red | 7 | 0.05 |  |  |  |  |
|  | White | 7 | 0.05 |  |  |  |  |
|  | Yellow | 22 | 0.16 |  |  |  |  |
| Presence of Epicauta sp. | No | 49 | 0.36 | 2.00 | 1.86 | 0.54 | 0.65 |
|  | Yes | 87 | 0.64 |  |  |  |  |
| Degree of dehiscence | Light | 103 | 0.76 | 3.00 | 1.59 | 0.63 | 0.59 |
|  | Regular | 32 | 0.24 |  |  |  |  |
|  |  | 1 | 0.01 |  |  |  |  |
| Lodging of the plant | No | 117 | 0.86 | 2.00 | 1.32 | 0.76 | 0.40 |
|  | Yes | 19 | 0.14 |  |  |  |  |
| Seed coat (episperm) color | Black | 4 | 0.03 | 9.00 | 3.07 | 0.33 | 1.45 |
|  | Cafe | 4 | 0.03 |  |  |  |  |
|  | Cafe claro | 3 | 0.02 |  |  |  |  |
|  | Cafe oscuro | 3 | 0.02 |  |  |  |  |
|  | Cafe rojizo | 12 | 0.09 |  |  |  |  |
|  | Cream | 66 | 0.49 |  |  |  |  |
|  | Transparent | 5 | 0.04 |  |  |  |  |
|  | White | 38 | 0.28 |  |  |  |  |
|  |  | 1 | 0.01 |  |  |  |  |
| Grain shape | Cilindrico | 107 | 0.79 | 4.00 | 1.55 | 0.65 | 0.67 |
|  | Ellipsoidal | 7 | 0.05 |  |  |  |  |
|  | Lenticular | 21 | 0.15 |  |  |  |  |
|  |  | 1 | 0.01 |  |  |  |  |
| Mean ± SE |  |  |  | 4.82 ± 0.90 | 2.39 ± 0.35 | 0.50 ± 0.06 | 0.97 ± 0.15 |

Code

```
#write_xlsx(Diversity, "manuscript/Table-1.xlsx")

Diversity %>% web_table()
```

Code

```
# Analisis de correlación 

cor_matrix <- Diversity %>% 
  dplyr::select(Na, fi,Nc, Ne, He, `H′`)

pairs.panels(cor_matrix,
             method = "spearman",   
             hist.col = "red",      
             pch = 21,             
             stars = T,        
             scale = F,
             lm = T)
```

# 7 Objective 2

## 7.1 Study of the morphological diversity of quantitative variables of quinoa accessions and variables with the greatest discriminant contribution

Code

```
quanti <- fb %>% 
  dplyr::select(c(1:sample, spad, ppfi,ndh50f,ndh50mf, lgtp, dtp, alp, dmt, palp10, ps10, p100g )) 
str(quanti)
## 'data.frame':    1030 obs. of  24 variables:
##  $ PLOT     : Factor w/ 162 levels "0011","1001",..: 5 5 5 5 5 6 6 6 6 6 ...
##  $ CODIGO   : Factor w/ 162 levels "CH1","CH2","CH3",..: 8 8 8 8 8 9 9 9 9 9 ...
##  $ NUM      : Factor w/ 159 levels "0011","10","100",..: 91 91 91 91 91 102 102 102 102 102 ...
##  $ EXPT     : Factor w/ 1 level "quinoaGB": 1 1 1 1 1 1 1 1 1 1 ...
##  $ LOCATION : Factor w/ 1 level "1": 1 1 1 1 1 1 1 1 1 1 ...
##  $ YEAR     : Factor w/ 1 level "2025": 1 1 1 1 1 1 1 1 1 1 ...
##  $ ROW      : Factor w/ 16 levels "1","10","11",..: 1 1 1 1 1 1 1 1 1 1 ...
##  $ COLUMN   : Factor w/ 13 levels "1","10","11",..: 8 8 8 8 8 9 9 9 9 9 ...
##  $ CHECKS   : Factor w/ 2 levels "0","1": 1 1 1 1 1 1 1 1 1 1 ...
##  $ BLOCK    : Factor w/ 16 levels "1","10","11",..: 1 1 1 1 1 1 1 1 1 1 ...
##  $ ENTRY    : Factor w/ 158 levels "1","10","100",..: 93 93 93 93 93 104 104 104 104 104 ...
##  $ TREATMENT: Factor w/ 158 levels "CH1","CH2","CH3",..: 93 93 93 93 93 104 104 104 104 104 ...
##  $ sample   : Factor w/ 5 levels "1","2","3","4",..: 1 2 3 4 5 1 2 3 4 5 ...
##  $ spad     : chr  "44.8" "43.3" "44.3" NA ...
##  $ ppfi     : chr  "100" NA NA NA ...
##  $ ndh50f   : chr  "76" NA NA NA ...
##  $ ndh50mf  : chr  "90" NA NA NA ...
##  $ lgtp     : chr  "30.5" "40" "30" "43" ...
##  $ dtp      : chr  "8.66" "8.14" "8.05" "6.3" ...
##  $ alp      : chr  "97.5" NA NA NA ...
##  $ dmt      : chr  "1.6" "1.33" "1" NA ...
##  $ palp10   : chr  "1.95" NA NA NA ...
##  $ ps10     : chr  "638.26" NA NA NA ...
##  $ p100g    : chr  "0.34" NA NA NA ...

exp <- quanti%>%
  mutate(geno = case_when(
    CHECKS == 0 ~ as.character(CODIGO)
    , CHECKS == 1 ~ "CHECKS"
  ), .after = CHECKS) %>% 
  mutate(across(c(1:sample), as.factor)) %>% 
  mutate(across(c(spad, ppfi,ndh50f,ndh50mf, lgtp, dtp, alp, dmt, palp10, ps10, p100g), as.numeric)) %>% 
  dplyr::select(1:sample, spad, ppfi,ndh50f,ndh50mf, lgtp, dtp, alp, dmt, palp10, ps10, p100g) %>% 
  
  # Hi
  mutate(hi = (ps10/((palp10 * 1000) + ps10)) *100) %>% 
  mutate(p100g = (p100g*10))

str(exp)
## 'data.frame':    1030 obs. of  26 variables:
##  $ PLOT     : Factor w/ 162 levels "0011","1001",..: 5 5 5 5 5 6 6 6 6 6 ...
##  $ CODIGO   : Factor w/ 162 levels "CH1","CH2","CH3",..: 8 8 8 8 8 9 9 9 9 9 ...
##  $ NUM      : Factor w/ 159 levels "0011","10","100",..: 91 91 91 91 91 102 102 102 102 102 ...
##  $ EXPT     : Factor w/ 1 level "quinoaGB": 1 1 1 1 1 1 1 1 1 1 ...
##  $ LOCATION : Factor w/ 1 level "1": 1 1 1 1 1 1 1 1 1 1 ...
##  $ YEAR     : Factor w/ 1 level "2025": 1 1 1 1 1 1 1 1 1 1 ...
##  $ ROW      : Factor w/ 16 levels "1","10","11",..: 1 1 1 1 1 1 1 1 1 1 ...
##  $ COLUMN   : Factor w/ 13 levels "1","10","11",..: 8 8 8 8 8 9 9 9 9 9 ...
##  $ CHECKS   : Factor w/ 2 levels "0","1": 1 1 1 1 1 1 1 1 1 1 ...
##  $ geno     : Factor w/ 159 levels "CHECKS","UNTRM121001",..: 5 5 5 5 5 6 6 6 6 6 ...
##  $ BLOCK    : Factor w/ 16 levels "1","10","11",..: 1 1 1 1 1 1 1 1 1 1 ...
##  $ ENTRY    : Factor w/ 158 levels "1","10","100",..: 93 93 93 93 93 104 104 104 104 104 ...
##  $ TREATMENT: Factor w/ 158 levels "CH1","CH2","CH3",..: 93 93 93 93 93 104 104 104 104 104 ...
##  $ sample   : Factor w/ 5 levels "1","2","3","4",..: 1 2 3 4 5 1 2 3 4 5 ...
##  $ spad     : num  44.8 43.3 44.3 NA NA 47.4 50.7 51 NA NA ...
##  $ ppfi     : num  100 NA NA NA NA 100 NA NA NA NA ...
##  $ ndh50f   : num  76 NA NA NA NA 76 NA NA NA NA ...
##  $ ndh50mf  : num  90 NA NA NA NA 90 NA NA NA NA ...
##  $ lgtp     : num  30.5 40 30 43 35 25 28.5 26.5 31 34 ...
##  $ dtp      : num  8.66 8.14 8.05 6.3 10.44 ...
##  $ alp      : num  97.5 NA NA NA NA 83.5 NA NA NA NA ...
##  $ dmt      : num  1.6 1.33 1 NA NA 0.79 1.1 0.87 NA NA ...
##  $ palp10   : num  1.95 NA NA NA NA 1.42 NA NA NA NA ...
##  $ ps10     : num  638 NA NA NA NA ...
##  $ p100g    : num  3.4 NA NA NA NA 2.9 NA NA NA NA ...
##  $ hi       : num  24.7 NA NA NA NA ...

exp %>% web_table()
```

### 7.1.1 Augmented design with entries as random effects and checks as fixed effects

The evaluated traits include panicle color at 50% flowering (cdpf), panicle color at 50% physiological maturity (cpmf), panicle shape (frp), panicle length (lgtp), panicle diameter (dtp), panicle density (dsp), days to 50% flowering (ndh50f), days to 50% physiological maturity (ndh50mf), degree of dehiscence (gdh), 1000-grain weight (p100g), biomass of 10 plants (palp10), seed weight from 10 plants (ps10), seed coat (episperm) color (cepp), chlorophyll content at 50% flowering (spad), lodging of the plant (acm), plant height (alp), stem diameter (dmt), growth habit (hbc), main stem color (ctp), grain shape (fmg), presence of Epicauta sp. (pse), incidence of Peronospora variabilis (ppfi), and harvest index (hi).

Model: y ~ 0 + (1|BLOCK) + (1|ROW) + (1|COLUMN) + (1|CODIGO:CHECKS) + geno

Code

```
rslt <- 15:length(exp) %>% map(\(x) {
  
trait <- names(exp)[x]

exp %>% 
  drop_na(trait) %>% 
  H2cal(trait = trait
    , gen.name = "geno"
    , rep.n = 3
    , fixed.model = ~ 0 + (1|BLOCK) + (1|ROW) + (1|COLUMN) + (1|CODIGO:CHECKS) + geno
    , random.model = ~ 1 + (1|BLOCK) + (1|ROW) + (1|COLUMN) + (1|CODIGO:CHECKS) + (1|geno)
    , summary = T
    , emmeans = F
    , plot_diag = T
    , outliers.rm = F
    )

})
## Linear mixed model fit by REML ['lmerMod']
## Formula: 
## spad ~ 1 + (1 | BLOCK) + (1 | ROW) + (1 | COLUMN) + (1 | CODIGO:CHECKS) +  
##     (1 | geno)
##    Data: dt.rm
## Weights: weights
## 
## REML criterion at convergence: 3389.2
## 
## Scaled residuals: 
##     Min      1Q  Median      3Q     Max 
## -4.3859 -0.5413 -0.0228  0.5663  2.6020 
## 
## Random effects:
##  Groups        Name        Variance       Std.Dev. 
##  CODIGO:CHECKS (Intercept) 16.68940663282 4.0852670
##  geno          (Intercept) 16.61154520441 4.0757263
##  ROW           (Intercept) 10.90918983138 3.3029063
##  BLOCK         (Intercept)  9.84747877729 3.1380693
##  COLUMN        (Intercept)  0.00000008373 0.0002894
##  Residual                  26.23810054405 5.1223140
## Number of obs: 513, groups:  
## CODIGO:CHECKS, 139; geno, 137; ROW, 16; BLOCK, 16; COLUMN, 13
## 
## Fixed effects:
##             Estimate Std. Error t value
## (Intercept)    59.05       1.27   46.51
## optimizer (nloptwrap) convergence code: 0 (OK)
## boundary (singular) fit: see help('isSingular')
```

```
## Linear mixed model fit by REML ['lmerMod']
## Formula: 
## ppfi ~ 1 + (1 | BLOCK) + (1 | ROW) + (1 | COLUMN) + (1 | CODIGO:CHECKS) +  
##     (1 | geno)
##    Data: dt.rm
## Weights: weights
## 
## REML criterion at convergence: 1627.2
## 
## Scaled residuals: 
##      Min       1Q   Median       3Q      Max 
## -2.36848 -0.38338  0.00052  0.48084  1.76248 
## 
## Random effects:
##  Groups        Name        Variance Std.Dev.
##  CODIGO:CHECKS (Intercept) 292.1020 17.0910 
##  geno          (Intercept) 138.7648 11.7798 
##  ROW           (Intercept) 220.2424 14.8406 
##  BLOCK         (Intercept)   0.3415  0.5844 
##  COLUMN        (Intercept)   0.0000  0.0000 
##  Residual                  369.1060 19.2121 
## Number of obs: 171, groups:  
## CODIGO:CHECKS, 139; geno, 137; ROW, 16; BLOCK, 16; COLUMN, 13
## 
## Fixed effects:
##             Estimate Std. Error t value
## (Intercept)   57.349      4.446    12.9
## optimizer (nloptwrap) convergence code: 0 (OK)
## boundary (singular) fit: see help('isSingular')
```

```
## Linear mixed model fit by REML ['lmerMod']
## Formula: 
## ndh50f ~ 1 + (1 | BLOCK) + (1 | ROW) + (1 | COLUMN) + (1 | CODIGO:CHECKS) +  
##     (1 | geno)
##    Data: dt.rm
## Weights: weights
## 
## REML criterion at convergence: 932.6
## 
## Scaled residuals: 
##     Min      1Q  Median      3Q     Max 
## -0.9375 -0.0836 -0.0436  0.0721  4.5142 
## 
## Random effects:
##  Groups        Name        Variance Std.Dev.
##  CODIGO:CHECKS (Intercept)  0.70343 0.8387  
##  geno          (Intercept) 29.15560 5.3996  
##  ROW           (Intercept)  0.01158 0.1076  
##  BLOCK         (Intercept)  0.06854 0.2618  
##  COLUMN        (Intercept)  0.03227 0.1796  
##  Residual                   0.34097 0.5839  
## Number of obs: 171, groups:  
## CODIGO:CHECKS, 139; geno, 137; ROW, 16; BLOCK, 16; COLUMN, 13
## 
## Fixed effects:
##             Estimate Std. Error t value
## (Intercept)  80.3458     0.4777   168.2
## optimizer (nloptwrap) convergence code: 0 (OK)
## unable to evaluate scaled gradient
## Model failed to converge: degenerate  Hessian with 1 negative eigenvalues
```

```
## Linear mixed model fit by REML ['lmerMod']
## Formula: 
## ndh50mf ~ 1 + (1 | BLOCK) + (1 | ROW) + (1 | COLUMN) + (1 | CODIGO:CHECKS) +  
##     (1 | geno)
##    Data: dt.rm
## Weights: weights
## 
## REML criterion at convergence: 300.4
## 
## Scaled residuals: 
##            Min             1Q         Median             3Q            Max 
## -0.00000038647 -0.00000018035 -0.00000002576  0.00000025765  0.00000046377 
## 
## Random effects:
##  Groups        Name        Variance                   Std.Dev.       
##  CODIGO:CHECKS (Intercept) 39.14778633300903720737551 6.2568191865363
##  geno          (Intercept) 54.24737881523813598505512 7.3652819915627
##  ROW           (Intercept)  0.00000000000000000000000 0.0000000000000
##  BLOCK         (Intercept)  0.00000000000000000000000 0.0000000000000
##  COLUMN        (Intercept)  0.00000000000000000003457 0.0000000001859
##  Residual                   0.00000000000760548190092 0.0000027578038
## Number of obs: 171, groups:  
## CODIGO:CHECKS, 139; geno, 137; ROW, 16; BLOCK, 16; COLUMN, 13
## 
## Fixed effects:
##             Estimate Std. Error t value
## (Intercept)   103.24       0.82   125.9
## optimizer (nloptwrap) convergence code: 0 (OK)
## boundary (singular) fit: see help('isSingular')
```

```
## Linear mixed model fit by REML ['lmerMod']
## Formula: 
## lgtp ~ 1 + (1 | BLOCK) + (1 | ROW) + (1 | COLUMN) + (1 | CODIGO:CHECKS) +  
##     (1 | geno)
##    Data: dt.rm
## Weights: weights
## 
## REML criterion at convergence: 5477
## 
## Scaled residuals: 
##     Min      1Q  Median      3Q     Max 
## -6.2176 -0.5301 -0.0382  0.5337  3.2277 
## 
## Random effects:
##  Groups        Name        Variance  Std.Dev.
##  CODIGO:CHECKS (Intercept)  0.759829 0.87168 
##  geno          (Intercept) 23.057330 4.80180 
##  ROW           (Intercept)  0.000469 0.02166 
##  BLOCK         (Intercept)  8.781491 2.96336 
##  COLUMN        (Intercept)  3.600673 1.89754 
##  Residual                  25.320018 5.03190 
## Number of obs: 855, groups:  
## CODIGO:CHECKS, 139; geno, 137; ROW, 16; BLOCK, 16; COLUMN, 13
## 
## Fixed effects:
##             Estimate Std. Error t value
## (Intercept)   27.137      1.022   26.54
## optimizer (nloptwrap) convergence code: 0 (OK)
## unable to evaluate scaled gradient
## Model failed to converge: degenerate  Hessian with 1 negative eigenvalues
```

```
## Linear mixed model fit by REML ['lmerMod']
## Formula: 
## dtp ~ 1 + (1 | BLOCK) + (1 | ROW) + (1 | COLUMN) + (1 | CODIGO:CHECKS) +  
##     (1 | geno)
##    Data: dt.rm
## Weights: weights
## 
## REML criterion at convergence: 3579.4
## 
## Scaled residuals: 
##     Min      1Q  Median      3Q     Max 
## -2.9533 -0.6008 -0.0970  0.5080  5.4615 
## 
## Random effects:
##  Groups        Name        Variance Std.Dev.
##  CODIGO:CHECKS (Intercept) 3.6433   1.9087  
##  geno          (Intercept) 0.5012   0.7080  
##  ROW           (Intercept) 0.1381   0.3717  
##  BLOCK         (Intercept) 0.8205   0.9058  
##  COLUMN        (Intercept) 0.3712   0.6093  
##  Residual                  2.5360   1.5925  
## Number of obs: 855, groups:  
## CODIGO:CHECKS, 139; geno, 137; ROW, 16; BLOCK, 16; COLUMN, 13
## 
## Fixed effects:
##             Estimate Std. Error t value
## (Intercept)   7.2380     0.3508   20.63
## optimizer (nloptwrap) convergence code: 0 (OK)
## unable to evaluate scaled gradient
## Model failed to converge: degenerate  Hessian with 1 negative eigenvalues
```

```
## Linear mixed model fit by REML ['lmerMod']
## Formula: 
## alp ~ 1 + (1 | BLOCK) + (1 | ROW) + (1 | COLUMN) + (1 | CODIGO:CHECKS) +  
##     (1 | geno)
##    Data: dt.rm
## Weights: weights
## 
## REML criterion at convergence: 1547.1
## 
## Scaled residuals: 
##      Min       1Q   Median       3Q      Max 
## -2.06239 -0.38378  0.02966  0.34881  1.76214 
## 
## Random effects:
##  Groups        Name        Variance Std.Dev.
##  CODIGO:CHECKS (Intercept)  19.28    4.391  
##  geno          (Intercept) 361.19   19.005  
##  ROW           (Intercept)  65.97    8.122  
##  BLOCK         (Intercept)  27.74    5.267  
##  COLUMN        (Intercept)  31.04    5.571  
##  Residual                  156.44   12.507  
## Number of obs: 171, groups:  
## CODIGO:CHECKS, 139; geno, 137; ROW, 16; BLOCK, 16; COLUMN, 13
## 
## Fixed effects:
##             Estimate Std. Error t value
## (Intercept)   110.81       3.51   31.57
```

```
## Linear mixed model fit by REML ['lmerMod']
## Formula: 
## dmt ~ 1 + (1 | BLOCK) + (1 | ROW) + (1 | COLUMN) + (1 | CODIGO:CHECKS) +  
##     (1 | geno)
##    Data: dt.rm
## Weights: weights
## 
## REML criterion at convergence: 126.4
## 
## Scaled residuals: 
##     Min      1Q  Median      3Q     Max 
## -2.3014 -0.5457 -0.0659  0.4803  4.0848 
## 
## Random effects:
##  Groups        Name        Variance Std.Dev.
##  CODIGO:CHECKS (Intercept) 0.004185 0.06469 
##  geno          (Intercept) 0.044871 0.21183 
##  ROW           (Intercept) 0.004584 0.06770 
##  BLOCK         (Intercept) 0.011081 0.10527 
##  COLUMN        (Intercept) 0.008321 0.09122 
##  Residual                  0.046391 0.21539 
## Number of obs: 513, groups:  
## CODIGO:CHECKS, 139; geno, 137; ROW, 16; BLOCK, 16; COLUMN, 13
## 
## Fixed effects:
##             Estimate Std. Error t value
## (Intercept)  1.14771    0.04595   24.98
## optimizer (nloptwrap) convergence code: 0 (OK)
## unable to evaluate scaled gradient
## Model failed to converge: degenerate  Hessian with 1 negative eigenvalues
```

```
## Linear mixed model fit by REML ['lmerMod']
## Formula: 
## palp10 ~ 1 + (1 | BLOCK) + (1 | ROW) + (1 | COLUMN) + (1 | CODIGO:CHECKS) +  
##     (1 | geno)
##    Data: dt.rm
## Weights: weights
## 
## REML criterion at convergence: 284.4
## 
## Scaled residuals: 
##      Min       1Q   Median       3Q      Max 
## -1.40159 -0.21111 -0.02786  0.16187  2.10940 
## 
## Random effects:
##  Groups        Name        Variance       Std.Dev.  
##  CODIGO:CHECKS (Intercept) 0.000000000202 0.00001421
##  geno          (Intercept) 0.413444760514 0.64299670
##  ROW           (Intercept) 0.019896777652 0.14105594
##  BLOCK         (Intercept) 0.004965857478 0.07046884
##  COLUMN        (Intercept) 0.014946582334 0.12225622
##  Residual                  0.025611251249 0.16003516
## Number of obs: 169, groups:  
## CODIGO:CHECKS, 139; geno, 137; ROW, 16; BLOCK, 16; COLUMN, 13
## 
## Fixed effects:
##             Estimate Std. Error t value
## (Intercept)  1.21969    0.07718    15.8
## optimizer (nloptwrap) convergence code: 0 (OK)
## boundary (singular) fit: see help('isSingular')
```

```
## Linear mixed model fit by REML ['lmerMod']
## Formula: 
## ps10 ~ 1 + (1 | BLOCK) + (1 | ROW) + (1 | COLUMN) + (1 | CODIGO:CHECKS) +  
##     (1 | geno)
##    Data: dt.rm
## Weights: weights
## 
## REML criterion at convergence: 2248.7
## 
## Scaled residuals: 
##      Min       1Q   Median       3Q      Max 
## -0.88660 -0.33952 -0.03837  0.20518  3.05938 
## 
## Random effects:
##  Groups        Name        Variance Std.Dev.
##  CODIGO:CHECKS (Intercept)     0.00   0.000 
##  geno          (Intercept) 48816.45 220.944 
##  ROW           (Intercept)  1573.10  39.662 
##  BLOCK         (Intercept)    32.89   5.735 
##  COLUMN        (Intercept)   386.03  19.648 
##  Residual                   6619.11  81.358 
## Number of obs: 168, groups:  
## CODIGO:CHECKS, 138; geno, 136; ROW, 16; BLOCK, 16; COLUMN, 13
## 
## Fixed effects:
##             Estimate Std. Error t value
## (Intercept)   424.11      23.28   18.21
## optimizer (nloptwrap) convergence code: 0 (OK)
## boundary (singular) fit: see help('isSingular')
```

```
## Linear mixed model fit by REML ['lmerMod']
## Formula: 
## p100g ~ 1 + (1 | BLOCK) + (1 | ROW) + (1 | COLUMN) + (1 | CODIGO:CHECKS) +  
##     (1 | geno)
##    Data: dt.rm
## Weights: weights
## 
## REML criterion at convergence: 233.2
## 
## Scaled residuals: 
##      Min       1Q   Median       3Q      Max 
## -1.95295 -0.24696 -0.02353  0.27949  1.93963 
## 
## Random effects:
##  Groups        Name        Variance Std.Dev.
##  CODIGO:CHECKS (Intercept) 0.021881 0.14792 
##  geno          (Intercept) 0.243530 0.49349 
##  ROW           (Intercept) 0.000000 0.00000 
##  BLOCK         (Intercept) 0.000000 0.00000 
##  COLUMN        (Intercept) 0.009512 0.09753 
##  Residual                  0.042698 0.20664 
## Number of obs: 168, groups:  
## CODIGO:CHECKS, 138; geno, 136; ROW, 16; BLOCK, 16; COLUMN, 13
## 
## Fixed effects:
##             Estimate Std. Error t value
## (Intercept)  3.12038    0.05488   56.86
## optimizer (nloptwrap) convergence code: 0 (OK)
## boundary (singular) fit: see help('isSingular')
```

```
## Linear mixed model fit by REML ['lmerMod']
## Formula: 
## hi ~ 1 + (1 | BLOCK) + (1 | ROW) + (1 | COLUMN) + (1 | CODIGO:CHECKS) +  
##     (1 | geno)
##    Data: dt.rm
## Weights: weights
## 
## REML criterion at convergence: 1203.3
## 
## Scaled residuals: 
##     Min      1Q  Median      3Q     Max 
## -2.3138 -0.5441  0.0586  0.5505  4.6903 
## 
## Random effects:
##  Groups        Name        Variance Std.Dev.
##  CODIGO:CHECKS (Intercept)  0.000   0.000   
##  geno          (Intercept)  0.000   0.000   
##  ROW           (Intercept)  1.438   1.199   
##  BLOCK         (Intercept)  9.915   3.149   
##  COLUMN        (Intercept)  2.950   1.718   
##  Residual                  67.752   8.231   
## Number of obs: 168, groups:  
## CODIGO:CHECKS, 138; geno, 136; ROW, 16; BLOCK, 16; COLUMN, 13
## 
## Fixed effects:
##             Estimate Std. Error t value
## (Intercept)   25.746      1.165   22.09
## optimizer (nloptwrap) convergence code: 0 (OK)
## boundary (singular) fit: see help('isSingular')
```

Code

```
tabsmr <- 1:length(rslt) %>% map(\(x) {
      rslt[[x]][["tabsmr"]] 
      }) %>% 
  bind_rows()

blues <- 1:length(rslt) %>% map(\(x) {
      rslt[[x]][["blues"]] %>% dplyr::select(!matches("smith.w"))
      }) %>% Reduce(function(...) merge(..., all = TRUE
                                        , by = c('geno')), .)

blups <- 1:length(rslt) %>% map(\(x) {
      rslt[[x]][["blups"]] %>% dplyr::select(!matches("smith.w"))
      }) %>% Reduce(function(...) merge(..., all = TRUE
                                        , by = c('geno')), .)

blues %>% web_table()
```

# 8 Multivariate Analysis using BLUEs

Code

```
info2 <- read_sheet(gs, sheet = "descriptor")

abbreviatio_sp <- info2$abbreviatio_sp

abbreviatio_en  <- info2$abbreviatio_en

nuevos_nombres <- setNames(abbreviatio_en , abbreviatio_sp)

nuevos_nombres_validos <- nuevos_nombres[names(nuevos_nombres) %in% colnames(blues)]

mapping_rename <- setNames(names(nuevos_nombres_validos), nuevos_nombres_validos)
mapping_rename
##        PL        PD        DF        DM       TSW        PB        SW      SPAD 
##    "lgtp"     "dtp"  "ndh50f" "ndh50mf"   "p100g"  "palp10"    "ps10"    "spad" 
##        PH        SD      IPER        HI 
##     "alp"     "dmt"    "ppfi"      "hi"

blues <- blues %>% rename(!!!mapping_rename)

blues <- blues %>% 
  slice(-1) %>%
mutate(geno = str_remove(geno, "^UNTRM12"))

mv <- blues%>% 
  column_to_rownames("geno") %>% 
  PCA(scale.unit = T, graph = F)

pcavar <- mv %>% 
  plot.PCA(choix="var"
           , title=""
           , autoLab = "y"
           ,  cex = 0.8
           , shadowtext = T)

pcaind <- mv %>% 
  plot.PCA(choix="ind"
           , habillage =10
           , title=""
           , autoLab = "y"
           , cex = 0.8
           , shadowtext = T
           , label = "ind"
           )

list(pcavar, pcaind) %>% 
  plot_grid(plotlist = ., nrow = 1, labels = "AUTO"
            , rel_heights = c(1, 1.1)
            )
```

Code

```
hcpc <- HCPC(mv, graph = FALSE)

tree <- \() {
  plot.HCPC(hcpc,
          choice = "map",
          legend = list(x = "topright",
                        cex = 0.6,
                        inset = 0.001,
                        box.lty = 0),
          draw.tree = FALSE)
  
}

plot <- plot_grid(pcavar, tree,
                  nrow = 1, labels = "auto")
p <- plot %>%
  ggsave2(plot = .
          , "manuscript/Figure-4.jpg"
          , units = "cm", width = 35, height = 20)

p %>% include_graphics()
```

Code

```
# Analisis de correlación 
cuanti_matrix <- blues%>% 
  dplyr::select(PL,PD, DF, DM, TSW, PB,SW  ,SPAD, PH , SD, IPER,HI )


grid <- \() {
  
  pairs.panels(cuanti_matrix,
             method = "spearman",   
             hist.col = "red",      
             pch = 21,             
             stars = T,        
             scale = F,
             lm = T)  
  
}

pg <- grid %>% 
  plot_grid()

plot <- ggsave(filename = "manuscript/Figure-5.jpg"
               , width = 30
               , height = 30
               , units = "cm")
```

# 9 Objective 3

Code

```
library(corrplot)

var <- get_pca_var(mv)

tmp <- tempfile(fileext = ".png")
ppi <- 300
png(tmp, width=4*ppi, height=12*ppi, res=ppi)
corrplot(var$cor, 
         method="number",
         tl.col="black", 
         tl.srt=45)
graphics.off()

pt1 <- png::readPNG(tmp) %>%
  grid::rasterGrob(interpolate = TRUE)

pt2 <- fviz_eig(mv, 
                addlabels=TRUE,
                hjust = 0.05,
                barfill="white",
                barcolor ="darkblue",
                linecolor ="red") + 
  ylim(0, 45) +  
  labs(
    title = "PCA - percentage of explained variances",
    y = "Variance (%)") +
  theme_minimal()

pt3 <- fviz_contrib(mv,
                     choice = "var", 
                     axes = 1, 
                     top = 10,
                     fill = "white",
                     color = "darkblue",
                     sort.val = "desc") +
  ylim(0, 25) + 
  labs(title = "Dim 1 - variables contribution")

pt4 <- fviz_contrib(mv,
                     choice = "var", 
                     axes = 2, 
                     top = 10,
                     fill = "white",
                     color = "darkblue",
                     sort.val = "desc") +
  ylim(0, 30) +  
  labs(title = "Dim 2 - variables contribution")

plot <- ggdraw(xlim = c(0.0, 1.0), ylim = c(0, 1.0)) +
  draw_plot(pt1,  width = 0.4, height = 1.3, x = 0.6, y = -0.16) +  
  draw_plot(pt2,  width = 0.6, height = 0.34, x = 0.03, y = 0.66) +
  draw_plot(pt3, width = 0.6, height = 0.34, x = 0.03, y = 0.33) + 
  draw_plot(pt4, width = 0.6, height = 0.34, x = 0.03, y = 0.0) +
  draw_plot_label(
    label = c("a", "b", "c", "d"),
    x = c(0.005, 0.005, 0.005, 0.65),
    y = c(0.999, 0.67, 0.34, 0.999))

print(plot)

pt <- ggsave2(plot = plot, "files/SFigure-1.jpg", height = 25, width = 35, units = "cm")

pt %>% include_graphics()
```

# 10 Table blues

Code

```
# info2 <- read_sheet(gs, sheet = "descriptor")
# 
# abbreviatio_sp <- info2$abbreviatio_sp
# 
# abbreviatio_en  <- info2$abbreviatio_en
# 
# nuevos_nombres1 <- setNames(abbreviatio_en , abbreviatio_sp)
# 
# nuevos_nombres_validos1 <- nuevos_nombres1[names(nuevos_nombres1) %in% colnames(quali)]
# 
# mapping_rename1 <- setNames(names(nuevos_nombres_validos1), nuevos_nombres_validos1)
# mapping_rename1
# 
# quali <- quali %>% rename(!!!mapping_rename1)
# quali
# # Leer la hoja y preparar 'codes'
# codes <- read_sheet(gs, sheet = "STable7") %>%
#   mutate(
#     codigo_original = str_remove(codigo_original, "-\\d+$"),
#     CODIGO          = str_remove(CODIGO, "-20\\d{2}$"),
#     idx_original    = str_extract(codigo_original, "\\d+$"),
#     idx_codigo      = str_extract(CODIGO, "\\d+$"),
#     CODIGO          = str_replace(CODIGO, "\\d+$", idx_original),
#     codigo_untrm    = as.character(codigo_untrm)
#   ) %>%
#   arrange(codigo_untrm) %>%
#   select(
#     codigo_untrm,
#     codigo_original,
#     CODIGO,
#     `Fecha de colección`,
#     País,
#     Departamento,
#     Provincia,
#     Distrito,
#     Localidad,
#     `Altitud (msnm)`
#   )
# 
# # Preparar DT a partir de blues
# DT <- blues %>%
#   mutate(
#     geno = paste0("UNTRM12", geno),                  # agregar prefijo
#     across(2:13, ~ ifelse(. < 0, 0, .), .names = "{.col}"),  # reemplazar negativos por 0
#     across(2:13, ~ round(., 2))                      # redondear columnas numéricas
#   )
# 
# # Hacer join y limpiar datos
# blues_codes <- codes %>%
#   left_join(
#     DT %>% mutate(geno = as.character(geno)),       # asegurar coincidencia como character
#     by = c("codigo_untrm" = "geno")
#   ) %>%
#   mutate(
#     across(where(is.numeric), ~ replace_na(., 0)),  # reemplazar NAs por 0
#     codigo_untrm = str_replace(codigo_untrm, "^UNTRM12", "UNTRM-367-")  # renombrar código
#   ) %>%
#   select(
#     `UNTRM code` = codigo_untrm,
#     `UNSCH code` = codigo_original,
#     `collection date` = `Fecha de colección`,
#     `country` = País,
#     `department` = Departamento,
#     `province` = Provincia,
#     `district` = Distrito,
#     `locality` = Localidad,
#     `altitude (masl)` = `Altitud (msnm)`,
#     everything(),
#     -CODIGO
#   )
# 
# quali_sel <- quali%>%
#   mutate(
#     CODIGO = str_replace(CODIGO, "^UNTRM12", "UNTRM-367-")
#   ) %>%
#   dplyr::select(
#     CODIGO,
#     PCF, PSH, PCM, PDE, GH, MSC, EPI, DD, LOD, SCC, GS
#   )
# 
# blues_codes_final <- blues_codes %>%
#   left_join(quali_sel, by = c("UNTRM code" = "CODIGO"))
# 
# # blues_codes_final%>% web_table()
# # write_xlsx(blues_codes_final, "files/STable1.xlsx")
```

Code

```
info3 <- read_sheet(gs, sheet = "descriptor")
abbreviatio_en  <- info3$abbreviatio_en
descriptors<- info3$descriptors

nuevos_nombres3 <- setNames(descriptors, abbreviatio_en)

nuevos_nombres_validos3 <- nuevos_nombres3[names(nuevos_nombres3) %in% colnames(blues)]

mapping_rename3 <- setNames(names(nuevos_nombres_validos3), nuevos_nombres_validos3)
mapping_rename3
##                       Panicle length (cm) 
##                                      "PL" 
##                     Panicle diameter (cm) 
##                                      "PD" 
##              Days to 50% flowering (days) 
##                                      "DF" 
## Days to 50% physiological maturity (days) 
##                                      "DM" 
##                     1000-grain weight (g) 
##                                     "TSW" 
##                 Biomass of 10 plants (kg) 
##                                      "PB" 
##             Seed weight from10 plants (g) 
##                                      "SW" 
##      Chlorophyll content at 50% flowering 
##                                    "SPAD" 
##                         Plant height (cm) 
##                                      "PH" 
##                        Stem diameter (cm) 
##                                      "SD" 
##   Incidence of Peronospora variabilis (%) 
##                                    "IPER" 
##                             Harvest index 
##                                      "HI"

blues <- blues %>% rename(!!!mapping_rename3)
blues
##     geno Chlorophyll content at 50% flowering
## 1   1002                             44.66145
## 2   1004                             41.15094
## 3   1005                             51.09629
## 4   1006                             44.34072
## 5   1007                             48.93889
## 6   1008                             64.73799
## 7   1009                             51.14087
## 8   1010                             53.02558
## 9   1011                             48.91838
## 10  1012                             47.15891
## 11  1013                             52.64087
## 12  1014                             48.33199
## 13  1015                             43.89075
## 14  1016                             59.87099
## 15  1017                             50.50522
## 16  1019                             53.76262
## 17  1020                             50.50555
## 18  1021                             55.45751
## 19  1022                             57.20555
## 20  1023                             60.43559
## 21  1024                             65.38323
## 22  1025                             65.69433
## 23  1026                             74.22584
## 24  1027                             70.42494
## 25  1028                             64.78594
## 26  1029                             59.29449
## 27  1030                             79.44586
## 28  1031                             71.93866
## 29  1033                             56.52782
## 30  1034                             54.22956
## 31  1035                             74.28832
## 32  1036                             82.63522
## 33  1037                             66.93795
## 34  1038                             75.74817
## 35  1039                             72.46979
## 36  1040                             77.72174
## 37  1041                             76.55507
## 38  1042                             70.90312
## 39  1043                             44.63316
## 40  1044                             55.10454
## 41  1045                             59.66099
## 42  1046                             45.52583
## 43  1047                             60.11136
## 44  1048                             54.98593
## 45  1049                             45.86114
## 46  1050                             54.04584
## 47  1051                             53.93865
## 48  1052                             43.17198
## 49  1053                             55.84584
## 50  1054                             52.27215
## 51  1055                             64.93091
## 52  1056                             55.84448
## 53  1057                             53.34538
## 54  1058                             58.73610
## 55  1059                             55.05743
## 56  1060                             60.80908
## 57  1061                             55.54571
## 58  1062                             62.03100
## 59  1063                             66.44571
## 60  1064                             66.27841
## 61  1065                             67.39043
## 62  1066                             58.56819
## 63  1067                             65.99880
## 64  1068                             62.61856
## 65  1069                             56.73501
## 66  1070                             59.18638
## 67  1071                             64.67919
## 68  1072                             58.65305
## 69  1073                             61.66835
## 70  1074                             57.93836
## 71  1075                             62.93045
## 72  1076                             57.41069
## 73  1077                             54.84675
## 74  1078                             59.90232
## 75  1079                             56.24196
## 76  1080                             56.97859
## 77  1081                             61.39721
## 78  1082                             61.37859
## 79  1083                             52.24196
## 80  1084                             53.98440
## 81  1085                             53.40752
## 82  1086                             53.90569
## 83  1087                             63.50479
## 84  1088                             51.13246
## 85  1089                             58.70767
## 86  1090                             43.01851
## 87  1091                             58.75184
## 88  1092                             55.29237
## 89  1093                             56.00767
## 90  1094                             63.17460
## 91  1095                             63.40003
## 92  1096                             65.24694
## 93  1097                             59.44783
## 94  1098                             55.78300
## 95  1100                             60.91154
## 96  1104                             52.60476
## 97  1106                             58.76121
## 98  1107                             56.19271
## 99  1108                             67.79181
## 100 1109                             58.72803
## 101 1110                             51.87940
## 102 1111                             53.80554
## 103 1112                             52.03887
## 104 1113                             60.76136
## 105 1115                             69.03808
## 106 1116                             62.85165
## 107 1117                             53.55438
## 108 1118                             56.48292
## 109 1119                             57.38621
## 110 1120                             59.97150
## 111 1121                             67.37150
## 112 1122                             58.78621
## 113 1123                             59.24958
## 114 1124                             62.86225
## 115 1125                             51.60759
## 116 1126                             57.38536
## 117 1127                             50.15020
## 118 1128                             68.00239
## 119 1129                             64.64364
## 120 1130                             63.03688
## 121 1131                             58.76302
## 122 1132                             62.30355
## 123 1133                             56.25218
## 124 1134                             59.46943
## 125 1135                             64.52819
## 126 1137                             53.94449
## 127 1138                             73.16672
## 128 1140                             67.14299
## 129 1145                             63.56650
## 130 1147                             69.09711
## 131 1148                             63.28354
## 132 1149                             67.82478
## 133 1150                             43.43332
## 134 1151                             65.11802
## 135 1155                             71.13493
## 136 1158                             37.45123
##     Incidence of Peronospora variabilis (%) Days to 50% flowering (days)
## 1                                        80                     74.03343
## 2                                       100                     75.90757
## 3                                       100                     75.86419
## 4                                        70                     83.86872
## 5                                        90                     75.86236
## 6                                        35                     84.90967
## 7                                        35                     83.90278
## 8                                        35                     89.91455
## 9                                        35                     83.90524
## 10                                       10                     83.91455
## 11                                       10                     89.90278
## 12                                       70                     75.79332
## 13                                      100                     76.01825
## 14                                       70                     84.05945
## 15                                       60                     84.01214
## 16                                       90                     78.01397
## 17                                       50                     94.01816
## 18                                       60                     76.08922
## 19                                       50                     84.01816
## 20                                       50                     78.02078
## 21                                       60                     75.99006
## 22                                       70                     75.99459
## 23                                       12                     83.98823
## 24                                       70                     94.03554
## 25                                       60                     83.76941
## 26                                       90                     84.02865
## 27                                       50                     94.04041
## 28                                       50                     84.03111
## 29                                      100                     76.02865
## 30                                      100                     75.77118
## 31                                       50                     83.99610
## 32                                        0                     84.03731
## 33                                       60                     89.99636
## 34                                       20                     84.03521
## 35                                       80                     77.99602
## 36                                      100                     76.06708
## 37                                       50                     78.06708
## 38                                       50                     75.99602
## 39                                       90                     73.99863
## 40                                        0                     90.05434
## 41                                        0                     90.01549
## 42                                      100                     76.00913
## 43                                       70                     78.01524
## 44                                       60                     83.79031
## 45                                      100                     84.04955
## 46                                       70                     78.06132
## 47                                      100                     76.05201
## 48                                       30                     94.05201
## 49                                       70                     84.06132
## 50                                      100                     75.76805
## 51                                       80                     75.99297
## 52                                      100                     78.03418
## 53                                       80                     83.98687
## 54                                       50                     75.98870
## 55                                       40                     76.03208
## 56                                        0                     83.99550
## 57                                       20                     73.99289
## 58                                       40                     76.06395
## 59                                       10                     83.99289
## 60                                       20                     84.06442
## 61                                       40                     84.02104
## 62                                       30                     78.02557
## 63                                       50                     84.06652
## 64                                       60                     74.02532
## 65                                       10                     78.05963
## 66                                       60                     84.07140
## 67                                       20                     90.06209
## 68                                       30                     76.07140
## 69                                       30                     84.05963
## 70                                       50                     83.76830
## 71                                        0                     83.99322
## 72                                       50                     76.03443
## 73                                       10                     83.99348
## 74                                       30                     75.98895
## 75                                       50                     73.99575
## 76                                       10                     73.99314
## 77                                       20                     78.06420
## 78                                       30                     89.99314
## 79                                       20                     83.99575
## 80                                        0                     74.05546
## 81                                       80                     76.01661
## 82                                       50                     94.01025
## 83                                       50                     84.05756
## 84                                       50                     75.79143
## 85                                       40                     84.05067
## 86                                      100                     76.05313
## 87                                        0                     78.05313
## 88                                       30                     76.06244
## 89                                       30                     76.05067
## 90                                        0                     75.77087
## 91                                        0                     83.99579
## 92                                        0                     90.03700
## 93                                       40                     77.98968
## 94                                       40                     77.99605
## 95                                       60                     89.99832
## 96                                       30                     74.04376
## 97                                       50                     76.00491
## 98                                       20                     75.99855
## 99                                       40                     84.04586
## 100                                      50                     84.03897
## 101                                     100                     74.05074
## 102                                      80                     76.04143
## 103                                      50                     76.04143
## 104                                      30                     84.03897
## 105                                      80                     84.01685
## 106                                     100                     74.05806
## 107                                     100                     76.01711
## 108                                      80                     74.01938
## 109                                     100                     76.01677
## 110                                     100                     76.08783
## 111                                     100                     76.08783
## 112                                     100                     76.01677
## 113                                      80                     78.01938
## 114                                      80                     78.05622
## 115                                     100                     78.01284
## 116                                      80                     76.01737
## 117                                     100                     76.01101
## 118                                     100                     90.01712
## 119                                      80                     89.79219
## 120                                      80                     76.06320
## 121                                      80                     76.05389
## 122                                      20                     76.06320
## 123                                      70                     84.05143
## 124                                     100                     75.78907
## 125                                      60                     76.01400
## 126                                     100                     76.01425
## 127                                      80                     78.00972
## 128                                      50                     76.01391
## 129                                      20                     77.99314
## 130                                      60                     90.03409
## 131                                      50                     83.99288
## 132                                      30                     83.76796
## 133                                     100                     74.02719
## 134                                      20                     84.03896
## 135                                      80                     75.99433
## 136                                     100                     75.99459
##     Days to 50% physiological maturity (days) Panicle length (cm)
## 1                                          97           23.378900
## 2                                          90           35.608408
## 3                                          90           29.010289
## 4                                         112           35.731277
## 5                                          90           31.515082
## 6                                         100           34.989855
## 7                                         112           37.017197
## 8                                         119           33.862938
## 9                                         119           33.946434
## 10                                        104           24.462938
## 11                                        119           15.197197
## 12                                         90           22.934114
## 13                                         90           19.487748
## 14                                        119           22.260655
## 15                                        112           43.525882
## 16                                         90           26.621088
## 17                                        119           27.359411
## 18                                         90           28.944552
## 19                                        112           35.459411
## 20                                        100           30.393192
## 21                                         90           30.968446
## 22                                         90           27.889434
## 23                                        112           30.473239
## 24                                        119           25.108012
## 25                                         90           34.881472
## 26                                         90           37.855354
## 27                                        119           34.321095
## 28                                        112           23.204591
## 29                                         90           21.255354
## 30                                         90           17.669364
## 31                                        112           25.362998
## 32                                        119           20.795905
## 33                                        119           23.677326
## 34                                        119           24.954458
## 35                                        100           26.294661
## 36                                         90           17.379802
## 37                                         97           29.779802
## 38                                         90           25.394661
## 39                                         90           24.428442
## 40                                        119           13.375040
## 41                                        119           30.797908
## 42                                         90           26.081713
## 43                                        104           28.183580
## 44                                        119           28.989946
## 45                                        119           33.763828
## 46                                        100           33.029569
## 47                                         97           33.113066
## 48                                        119           27.713066
## 49                                        112           31.229569
## 50                                         90           22.991804
## 51                                         97           23.785438
## 52                                         97           22.518344
## 53                                        104           27.483571
## 54                                         90           25.678778
## 55                                        100           25.476898
## 56                                        119           34.350882
## 57                                        104           29.317101
## 58                                         97           25.702242
## 59                                        100           31.417101
## 60                                        119           27.629482
## 61                                        112           32.731363
## 62                                         97           43.152351
## 63                                        119           26.470929
## 64                                         90           25.138022
## 65                                        112           37.318271
## 66                                        112           27.484012
## 67                                        112           20.967508
## 68                                        100           21.184012
## 69                                        119           21.918271
## 70                                        119           16.573985
## 71                                        119           29.367618
## 72                                        100           23.700525
## 73                                        119           22.581947
## 74                                        100           25.160958
## 75                                         90           24.333063
## 76                                        100           26.499281
## 77                                        100           22.884422
## 78                                        119           26.299281
## 79                                        100           17.733063
## 80                                         90           23.686966
## 81                                         90           25.609834
## 82                                        119           22.093639
## 83                                        112           22.528412
## 84                                         90           21.801872
## 85                                        119           21.975754
## 86                                         90           23.524992
## 87                                         97           26.124992
## 88                                        100           22.441495
## 89                                         97           19.575754
## 90                                        100           18.447946
## 91                                        100           24.241579
## 92                                        112            9.134486
## 93                                        112           19.139713
## 94                                        112           21.955908
## 95                                        119           23.907024
## 96                                         97           34.426423
## 97                                        100           30.949291
## 98                                        100           33.433096
## 99                                        104           42.467869
## 100                                       100           31.515211
## 101                                        90           32.980952
## 102                                        97           29.864448
## 103                                       100           36.264448
## 104                                       112           32.715211
## 105                                       104           27.195397
## 106                                        97           23.928304
## 107                                       100           32.009726
## 108                                        97           23.160842
## 109                                        97           25.927060
## 110                                        97           25.312201
## 111                                        97           25.312201
## 112                                        97           25.527060
## 113                                        97           28.160842
## 114                                        97           25.212943
## 115                                       112           27.714823
## 116                                       100           30.335811
## 117                                        90           25.419617
## 118                                       119           32.921483
## 119                                       119           33.127849
## 120                                        97           27.467472
## 121                                        90           24.250969
## 122                                       100           24.567472
## 123                                       112           38.101731
## 124                                        97           26.303029
## 125                                        97           21.896663
## 126                                       100           20.910991
## 127                                       100           36.890003
## 128                                       100           28.528326
## 129                                       100           31.307015
## 130                                       119           25.325593
## 131                                       119           28.092686
## 132                                       112           41.499053
## 133                                        90           21.072935
## 134                                       112           33.138676
## 135                                        90           22.687440
## 136                                        90           23.501769
##     Panicle diameter (cm) Plant height (cm) Stem diameter (cm)
## 1                6.811941          82.39838         0.83962614
## 2                9.158028         101.19598         1.44554605
## 3               12.040786          82.08472         0.85328841
## 4               14.873914         134.06466         1.09194614
## 5               10.514854         128.26229         1.30874510
## 6                9.268559         145.28721         1.34588631
## 7               12.405953         114.41588         1.28527722
## 8                9.320064          90.08851         1.04011921
## 9                6.464282         151.26260         1.21789452
## 10               7.820064          80.68851         1.07345255
## 11               3.805953          92.61588         0.92527722
## 12               4.905665          68.60652         0.44578562
## 13               4.073596          49.28713         0.09888131
## 14               5.806195         135.39609         0.76617302
## 15               7.052490         109.27117         0.51569848
## 16               6.478422          83.19360         0.18357513
## 17               4.990455          88.08117         0.76543812
## 18               8.105964         111.14625         0.78598941
## 19              10.690455         128.78117         1.16543812
## 20               6.701590         121.39337         1.00586359
## 21               8.121053         153.35420         1.30895393
## 22               6.954181         104.33414         1.03761166
## 23               6.995121         154.43177         1.09107729
## 24               6.648826         149.55669         1.36821850
## 25               7.348296         127.76712         1.40783109
## 26               7.986220         144.18536         1.56427607
## 27               7.600331         156.95799         1.63578473
## 28               7.044548         119.03208         1.20689338
## 29               5.186220          86.68536         0.79427607
## 30               3.928559          59.24011         0.97407504
## 31               5.556490         117.42073         0.49717073
## 32               6.329088         175.02969         1.59446245
## 33               6.534444         124.30713         1.69385561
## 34               6.200558         110.93845         1.38412219
## 35               7.413349          82.91476         1.23706088
## 36               6.928858          81.77984         0.86094550
## 37              10.928858         121.77984         1.40094550
## 38              11.013349          91.91476         1.17706088
## 39               6.824483          56.22696         0.92081968
## 40               3.440704         113.11974         1.25267304
## 41               6.174590         168.48842         1.71573979
## 42               5.315529         101.08606         0.76253875
## 43               6.736636         117.60202         1.08905491
## 44               7.168704         127.02140         1.25262589
## 45              10.106628         130.83964         1.48573754
## 46              10.920740         124.81227         1.20724620
## 47               7.464957         123.68636         1.28168818
## 48               6.864957         143.68636         1.29168818
## 49               4.920740         134.61227         1.03724620
## 50               5.603179          86.43049         1.24878971
## 51               6.571111         105.11111         0.96188540
## 52               5.303709          93.72007         1.49251045
## 53               7.150004         128.59515         1.29870258
## 54               6.575937         107.51757         1.29657922
## 55               9.475179          98.42883         1.31550353
## 56               8.199104         146.71734         1.72886768
## 57              10.787970         112.40514         1.45844222
## 58               7.403478         115.97022         1.37232683
## 59               6.987970         127.40514         1.33510888
## 60               7.256734         120.71569         1.33957893
## 61               9.057493         145.60444         1.13065462
## 62               8.690620         108.88437         0.99264568
## 63               5.885265         134.80693         1.15325252
## 64               5.852667         106.19797         0.67929414
## 65               5.222659         120.13560         1.15931010
## 66               6.836770         101.20823         1.22748542
## 67               4.680988         109.28232         1.35192740
## 68               4.336770          71.10823         0.72415209
## 69               4.422659         114.43560         1.11931010
## 70               3.116088          84.05249         1.36409475
## 71               4.784019         146.23310         1.25052378
## 72               5.116618         121.84206         0.88448216
## 73               3.321973          82.11950         1.05387532
## 74               4.288845          86.23957         0.94188426
## 75               5.012013          90.83933         1.02417272
## 76               6.600878          94.12714         1.02708059
## 77               8.516387         110.59222         1.09429854
## 78               6.300878         128.52714         1.37374726
## 79               4.612013         117.83933         1.64083939
## 80              11.416391          90.65243         1.08192879
## 81               5.050277          97.22111         1.02832887
## 82               4.491216         118.61874         1.51846117
## 83               6.244921         123.74366         1.09226904
## 84               5.244391         107.95409         1.39521497
## 85               3.582316         114.37233         1.21499329
## 86               2.740644          64.21905         0.79427726
## 87               5.540644          90.21905         1.20094393
## 88               3.496427          92.14496         1.56316862
## 89               6.382316          94.37233         0.95499329
## 90               7.694436          78.21032         0.98527478
## 91               6.762367         107.39094         0.74837047
## 92               2.894966         144.99990         1.19566218
## 93               5.341261          95.37498         1.43185431
## 94               5.500321          96.27734         1.01838868
## 95               7.290361         137.99717         1.29868608
## 96              15.800484         100.71858         1.16087270
## 97               6.834370         117.08726         1.56393946
## 98               6.675309         146.68490         1.28740509
## 99               9.829014         158.80982         1.35454629
## 100              9.766408         170.43848         1.79727054
## 101              7.080519          88.21111         0.88211253
## 102              7.224737         116.28520         0.98988784
## 103              8.024737         113.28520         1.37322118
## 104              8.966408         166.43848         1.46727054
## 105              6.074395         156.97839         1.37263563
## 106              5.306993         115.58735         1.08992735
## 107              6.812349         116.86480         1.36265384
## 108              8.702388         103.58463         0.90961792
## 109              6.091254          98.27243         0.95252578
## 110              9.006763         112.33751         0.99307707
## 111              5.806763         127.33751         1.13641040
## 112              7.691254          98.27243         1.21919245
## 113              7.702388         114.58463         1.59628458
## 114              6.710416          85.58748         1.01526851
## 115              6.911174          87.47622         0.76634421
## 116              8.944302         103.95616         0.98166860
## 117              8.385242          74.55380         0.78846756
## 118             10.406348          92.06976         0.87165039
## 119             13.338417         128.88914         1.30188803
## 120              8.890452          98.08001         1.07984168
## 121              6.834670         105.15410         1.27095032
## 122              8.890452          99.08001         1.44984168
## 123             13.876341         122.30738         1.22833302
## 124              6.355710          70.71979         1.28142325
## 125              6.623642         109.90040         1.13118560
## 126              5.461596          88.78681         0.85787048
## 127              9.128468         106.30687         1.20921276
## 128              9.440501         118.19444         1.31440908
## 129              6.272443         138.38854         1.51109452
## 130             10.267087         129.11110         1.57503470
## 131             10.934489         117.50214         0.99107631
## 132              8.766558         142.32153         1.85464729
## 133              4.704482          84.73977         0.92775894
## 134             10.618593         125.51239         1.50593427
## 135              7.607873          80.88066         0.74267469
## 136              6.945827          87.76706         0.81269290
##     Biomass of 10 plants (kg) Seed weight from10 plants (g)
## 1                  0.63806610                    102.412421
## 2                  1.95623005                    697.456658
## 3                  1.28516210                    157.778879
## 4                  1.81755451                    578.364620
## 5                  0.67332599                    312.520886
## 6                  1.61173102                    635.505555
## 7                  1.44704552                    651.601954
## 8                  1.30054210                    468.423143
## 9                  1.67248288                    768.596518
## 10                 1.17054210                    397.593143
## 11                 1.14704552                    384.621954
## 12                 0.25732075                     95.151581
## 13                 0.04379588                     57.546227
## 14                 1.17857485                    257.382763
## 15                 1.09016982                    509.678094
## 16                 0.21200593                    145.256087
## 17                 1.15586129                    495.391191
## 18                 1.19984811                    433.493529
## 19                 1.77586129                    864.891191
## 20                 1.89434918                    699.731593
## 21                 1.15552208                    528.746671
## 22                 0.59791450                    189.552413
## 23                 2.22368598                    886.638678
## 24                 1.87209101                    507.163348
## 25                 1.04083690                    511.502165
## 26                 1.26740550                    488.379747
## 27                 2.02090209                    558.420936
## 28                 1.13284287                    393.144311
## 29                 0.52740550                     47.649747
## 30                 0.70273490                    407.232074
## 31                 0.86921003                    415.176720
## 32                 1.35398901                    446.903256
## 33                 1.53981250                    459.852322
## 34                 1.26848803                    298.984359
## 35                 0.32127545                    289.891684
## 36                 0.30526226                    103.144022
## 37                 1.14526226                    435.824022
## 38                -0.01872455                    113.341684
## 39                 0.24976334                     52.262086
## 40                 1.60752269                    391.787922
## 41                 1.56884715                    300.165885
## 42                 0.50461863                    175.032150
## 43                 0.62824469                    382.610283
## 44                 2.72176955                    998.215637
## 45                 1.89833815                    602.103218
## 46                 1.25183474                    540.744408
## 47                 1.42377552                    549.127783
## 48                 1.42377552                    406.757783
## 49                 1.21183474                    526.944408
## 50                 0.32531789                    211.513873
## 51                 0.62179302                    378.858519
## 52                 0.88657200                    301.975055
## 53                 1.70816696                    539.980386
## 54                 1.25000307                    562.238379
## 55                 0.98107102                    515.126158
## 56                 2.34234632                    687.173885
## 57                 1.61385843                    870.343483
## 58                 1.30784525                    494.585821
## 59                 0.68385843                    517.863483
## 60                 2.41010938                    569.082635
## 61                 1.68904142                    769.094856
## 62                 1.22143384                    396.730598
## 63                 1.62561035                    427.051532
## 64                 0.51083138                    373.854996
## 65                 1.75092484                    716.627931
## 66                 1.46442143                    362.919120
## 67                 1.80636221                    604.802495
## 68                 0.40442143                    118.719120
## 69                 0.91092484                    196.517931
## 70                 0.74414495                    181.728517
## 71                 2.26062008                    795.953163
## 72                 0.88539906                    272.289699
## 73                 1.51122255                    609.628765
## 74                 1.79883013                    689.543023
## 75                 0.98117339                    660.458529
## 76                 0.74268549                    556.848126
## 77                 2.48667231                    975.740465
## 78                 2.24268549                    818.438126
## 79                 1.99117339                    651.778529
## 80                 1.16624529                    357.574182
## 81                 0.80756975                    271.432144
## 82                 2.54334123                    830.778409
## 83                 0.80174626                    338.823079
## 84                 1.03049216                    467.171896
## 85                 1.35706075                    402.929478
## 86                 0.23249812                     55.854042
## 87                 1.74249812                    698.304042
## 88                 0.85055734                    398.390667
## 89                 0.31706075                     39.739478
## 90                -0.01412952                    -88.652638
## 91                 2.06234562                    850.512008
## 92                 0.86712459                     16.508544
## 93                 1.38871956                    227.603875
## 94                 1.73294808                    547.067610
## 95                 1.28289892                    225.047374
## 96                 1.87474083                    716.880850
## 97                 1.49606530                    483.148813
## 98                 2.91183677                   1007.275078
## 99                 1.55024181                    680.729747
## 100                1.92555630                    615.166146
## 101                0.94905288                    402.397336
## 102                1.89099366                    581.440711
## 103                1.34099366                    463.480711
## 104                1.53555630                    369.826146
## 105                1.83648568                    254.674293
## 106                0.63126465                    221.480829
## 107                0.91708814                    232.309895
## 108                0.41703898                    137.709659
## 109                0.15855109                    288.639257
## 110                0.57253790                    244.921595
## 111                1.28253790                    572.831595
## 112                0.03855109                    213.619257
## 113                1.25703898                    526.959659
## 114                0.70562372                    293.959464
## 115                0.86455577                            NA
## 116                1.39694818                    442.677427
## 117                0.31271966                     79.503692
## 118                1.34634572                    387.911825
## 119                2.88987059                    610.087179
## 120                1.10993577                    366.875949
## 121                1.14187655                    493.519324
## 122                0.58993577                    298.375949
## 123                1.77643918                    421.274760
## 124                0.11523699                     34.922817
## 125                0.54171212                    349.787464
## 126                0.52231459                     83.623065
## 127                1.27992217                    610.947323
## 128                0.66377753                    404.982427
## 129                2.38447000                    849.519371
## 130                2.06864651                    466.230306
## 131                1.21386753                    550.223770
## 132                3.41739240                   1091.179123
## 133                0.33396100                     16.406705
## 134                2.20745758                    460.507894
## 135                0.15878810                     32.074782
## 136                0.42939057                     -1.449617
##     1000-grain weight (g) Harvest index
## 1                     2.3      9.651909
## 2                     3.4     38.107850
## 3                     2.9     24.289533
## 4                     3.5     36.810772
## 5                     4.3     33.277076
## 6                     3.1     36.181530
## 7                     2.1     34.731290
## 8                     3.5     33.820267
## 9                     3.8     36.847491
## 10                    3.0     32.396680
## 11                    2.9     28.187585
## 12                    4.0     22.446068
## 13                    3.5     22.958297
## 14                    2.5     17.354550
## 15                    3.4     27.009624
## 16                    4.0     39.098994
## 17                    3.5     24.073322
## 18                    4.3     29.055126
## 19                    2.8     28.349104
## 20                    4.1     29.313782
## 21                    3.0     36.622422
## 22                    3.5     28.845506
## 23                    4.0     23.180927
## 24                    2.7     20.258323
## 25                    2.7     33.729639
## 26                    3.6     23.550290
## 27                    3.2     20.577347
## 28                    3.1     22.804358
## 29                    2.5      0.108843
## 30                    3.8     36.506780
## 31                    3.3     32.505775
## 32                    3.4     24.879405
## 33                    3.2     27.583260
## 34                    2.6     22.031502
## 35                    3.7     28.233931
## 36                    3.3     24.484146
## 37                    2.9     30.508812
## 38                    4.2     20.619961
## 39                    2.2     13.748591
## 40                    2.8     25.790241
## 41                    3.0     22.980603
## 42                    2.7     22.555296
## 43                    3.4     38.198073
## 44                    2.7     33.714388
## 45                    2.8     22.427531
## 46                    2.8     33.276230
## 47                    2.5     28.154224
## 48                    3.3     22.063418
## 49                    2.9     33.426962
## 50                    2.7     37.998641
## 51                    4.4     40.859794
## 52                    3.4     30.996322
## 53                    3.0     24.329378
## 54                    4.0     42.193386
## 55                    2.8     43.894919
## 56                    3.4     31.120271
## 57                    2.6     36.495309
## 58                    3.1     35.694459
## 59                    3.2     37.786470
## 60                    3.8     25.571708
## 61                    2.7     40.734026
## 62                    2.6     32.783573
## 63                    2.9     23.606624
## 64                    2.9     42.050516
## 65                    2.9     27.627165
## 66                    3.1     22.658201
## 67                    3.5     25.190856
## 68                    2.6     29.645388
## 69                    3.3     17.638873
## 70                    3.0     27.327351
## 71                    3.3     37.524187
## 72                    2.8     30.742229
## 73                    2.9     41.138076
## 74                    4.1     41.981714
## 75                    2.8     50.435420
## 76                    3.9     40.075764
## 77                    3.8     39.624799
## 78                    2.9     32.609104
## 79                    4.7     35.510947
## 80                    3.3     27.789680
## 81                    2.2     31.433506
## 82                    2.8     20.486386
## 83                    2.5     32.237216
## 84                    2.8     33.290297
## 85                    3.6     19.481203
## 86                    2.9     22.671363
## 87                    2.9     26.715214
## 88                    2.5     33.767724
## 89                    3.2     11.015741
## 90                    2.7     20.689348
## 91                    3.3     34.088375
## 92                    0.8     13.355941
## 93                    4.1     14.498139
## 94                    3.3     31.658360
## 95                    2.3     23.446555
## 96                    3.4     37.172043
## 97                    3.6     34.861741
## 98                    3.4     26.523773
## 99                    3.4     36.701706
## 100                   2.3     25.185585
## 101                   3.1     36.236037
## 102                   2.8     26.177910
## 103                   3.6     28.730944
## 104                   2.7     20.120976
## 105                   3.1     15.099941
## 106                   1.9     28.890835
## 107                   2.3     24.753105
## 108                   3.8     28.235555
## 109                   2.9     33.404240
## 110                   2.5     32.453116
## 111                   2.8     33.611214
## 112                   2.9     31.802686
## 113                   3.1     32.319292
## 114                   2.4     31.596679
## 115                    NA            NA
## 116                   3.4     27.313298
## 117                   4.0     14.200466
## 118                   3.4     23.201401
## 119                   2.9     19.480374
## 120                   3.2     23.325590
## 121                   3.2     26.598003
## 122                   3.8     34.596703
## 123                   3.5     12.605038
## 124                   2.8     16.636878
## 125                   2.5     25.635188
## 126                   2.7     13.813272
## 127                   2.7     25.180414
## 128                   3.3     15.560471
## 129                   3.0     15.972406
## 130                   2.4      3.339077
## 131                   2.5     18.835203
## 132                   3.6     12.426953
## 133                   3.2      7.970073
## 134                   2.9      2.046253
## 135                   3.6     13.873832
## 136                   3.0      9.954228

rl <- blues %>%
  dplyr::select(c(2:13)) %>%
  mutate(across(everything(), ~ifelse(.x < 0, 0, .x))) %>%
  summarise(across(
    everything(),
    list(
      Mean = ~round(mean(.x, na.rm = TRUE), 2),
      Min  = ~round(min(.x, na.rm = TRUE), 2),
      Max  = ~round(max(.x, na.rm = TRUE), 2),
      `CV(%)` = ~round(sd(.x, na.rm = TRUE) / mean(.x, na.rm = TRUE) * 100, 2)
    )
  )) %>%
  pivot_longer(
    cols = everything(),
    names_to = c("Quantitative trait", "stat"),
    names_sep = "_",
    values_to = "value"
  ) %>%
  pivot_wider(
    names_from = stat,
    values_from = value
  ) %>%
  mutate(across(Min:`CV(%)`, ~formatC(.x, format = "f", digits = 2))) 

rl%>% web_table()
```

Code

```
# write_xlsx(rl, "manuscript/Table-2.xlsx")
```
