# Supplementary material for: Agromorphological Characterization of Quinoa (Chenopodium quinoa Willd.) Under Andean–Amazonian Region of Peru
Source: Plants (Basel). 2025 Dec 4;14(23):3689. doi: 10.3390/plants14233689 (PMC12693800; doi:10.3390/plants14233689)
Supplement: Supplementary file 1 [file plants-14-03689-s001.zip › ESM_2.pdf]

Agromorphological characterization of quinoa (*Chenopodium quinoa* Willd.) under Andean–Amazonian region of Peru

Victor-Hugo Baldera-Chapoñan[1*]; Germán De la Cruz[4]; Segundo Oliva-Cruz[1,2,3]; Flavio Lozano-Isla[1,2,3*]

[1]*Facultad de Ingeniería y Ciencias Agrarias, Universidad Nacional Toribio Rodríguez de Mendoza de Amazonas (UNTRM), Amazonas, Perú.*

[2]*Centro de Investigación e Innovación en Granos y Semillas, Universidad Nacional Toribio Rodríguez de Mendoza de Amazonas (UNTRM), Amazonas, Perú.*

[3]*Instituto de Investigación para el Desarrollo Sustentable de Ceja de Selva, Universidad Nacional Toribio Rodríguez de Mendoza de Amazonas (UNTRM), Amazonas 01000, Perú.*

[4]*Facultad de Ciencias Agrícolas, Universidad Nacional de San Cristóbal de Huamanga, Ayacucho 05000, Perú.*

* Corresponding Authors: 7577995921@untrm.edu.pe; flavio.lozano@untrm.edu.pe

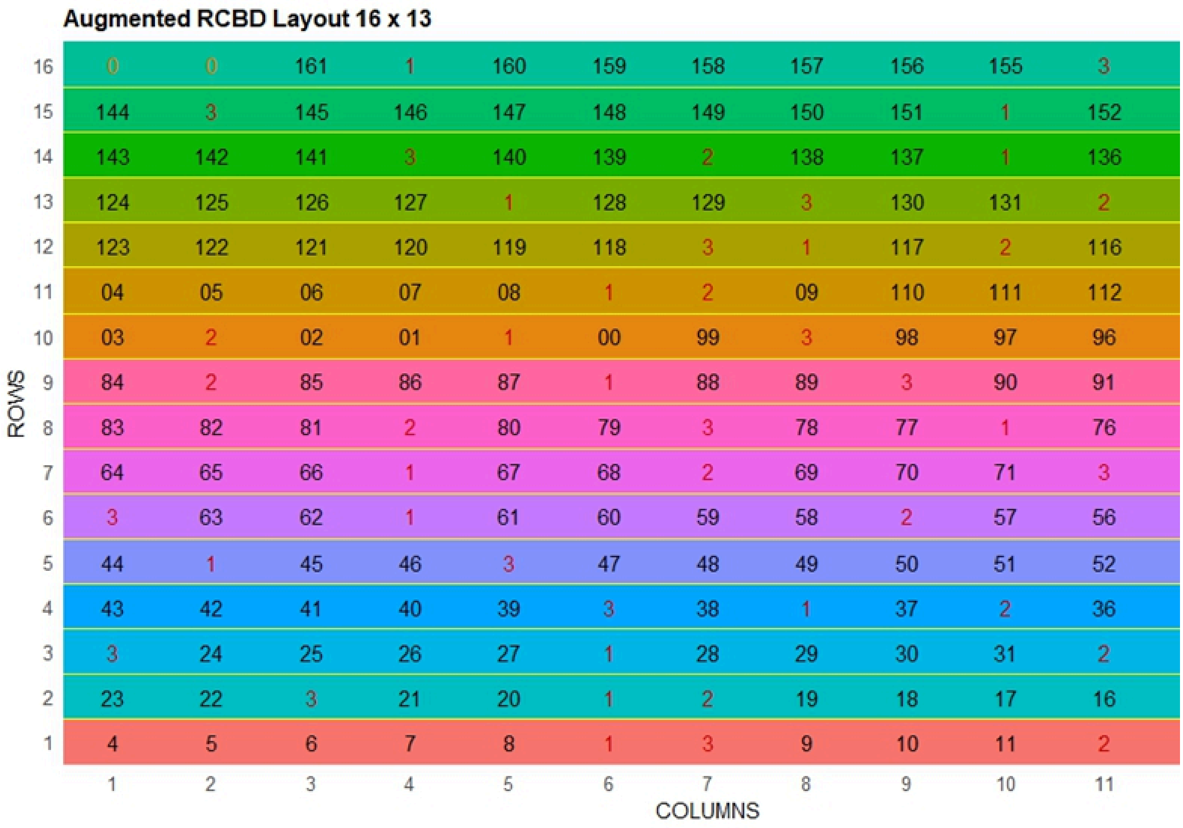

Supplementary Figure S1: Complete layout of the trial under the augmented design used to characterize 161 quinoa accessions (*Chenopodium quinoa* Willd.) in the Amazonas region, Peru. The diagram represents the full experimental matrix of 16 rows × 13 columns, where each row corresponds to one of the 16 blocks statistically defined by FieldHub. Codes “1,” “2,” and “3” denote the replicated check cultivars (INIA 415 Pasankalla, INIA 420 Negra Collana, and Blanca Juli, respectively), used as internal reference standards for comparing the non-replicated accessions. Numbers 4 to 164 identify the non-replicated accessions distributed across the 16 blocks of the augmented design.

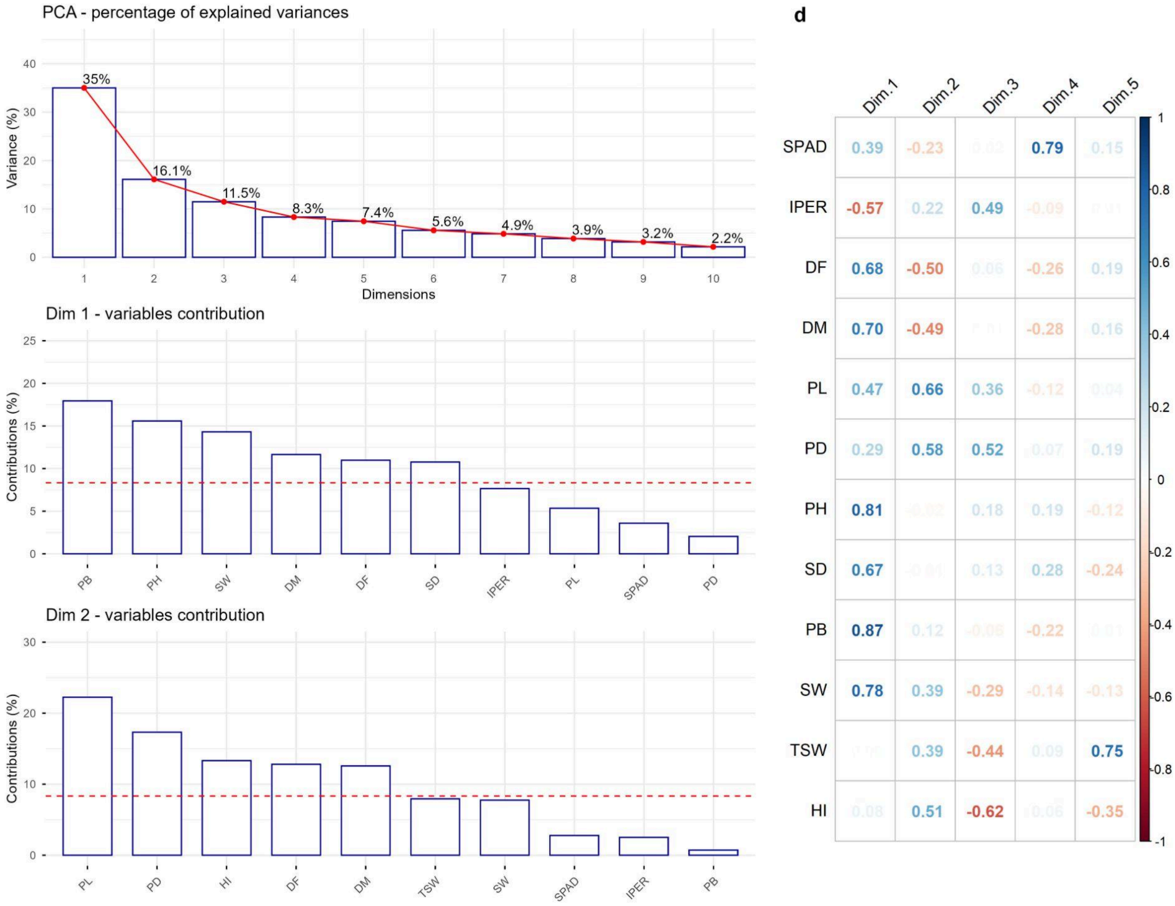

Supplementary Figure S2: The Principal Component Analysis (PCA) of the quinoa accessions summarized the multivariate structure of the agronomic traits. (a) Percentage of explained variance. (b) Dimension 1 variables contribution. (c) Dimension 2 variables contribution. (d) Correlation matrix between variables and dimensions. Where; panicle length (PL, cm), panicle diameter (PD, cm), number of days to 50% flowering (DF, days), number of days to 50% physiological maturity (DM, days), 1000-grain weight (TSW, g), biomass of 10 plants (PB, kg), seed weight from 10 plants (SW, g), chlorophyll content at 50% flowering (SPAD units), plant height (PH, cm), stem diameter (SD, mm), downy mildew severity caused by Peronospora variabilis (IPER, %), and harvest index (HI)
